# ZNF765 is a prognostic biomarker of hepatocellular carcinoma associated with cell cycle, immune infiltration, m^6^A modification, and drug susceptibility

**DOI:** 10.18632/aging.204827

**Published:** 2023-07-04

**Authors:** Yongqi Ding, Yiyang Gong, Hong Zeng, Gelin Song, Zichuan Yu, Bidong Fu, Yue Liu, Da Huang, Yanying Zhong

**Affiliations:** 1 Second Affiliated Hospital of Nanchang University, Nanchang, China; 2 Second College of Clinical Medicine, Nanchang University, Nanchang, China; 3 Department of Thyroid Surgery, Second Affiliated Hospital of Nanchang University, Nanchang, China; 4 Department of Obstetrics and Gynecology, Second Affiliated Hospital of Nanchang University, Nanchang, China

**Keywords:** hepatocellular carcinoma, ZNF765, biomarker, prognosis, immune infiltration

## Abstract

Hepatocellular carcinoma (HCC) is an ongoing challenge worldwide. Zinc finger protein 765 (ZNF765) is an important zinc finger protein that is related to the permeability of the blood-tumor barrier. However, the role of ZNF765 in HCC is unclear. This study evaluated the expression of ZNF765 in hepatocellular carcinoma and the impact of its expression on patient prognosis based on The Cancer Genome Atlas (TCGA). Immunohistochemical assays (IHC) were used to examine protein expression. Besides, a colony formation assay was used to examine cell viability. We also explored the relationship between ZNF765 and chemokines in the HCCLM3 cells by qRT-PCR. Moreover, we examined the effect of ZNF765 on cell resistance by measurement of the maximum half-inhibitory concentration. Our research revealed that ZNF765 expression in HCC samples was higher than that in normal samples, whose upregulation was not conducive to the prognosis. The results of GO, KEGG, and GSEA showed that ZNF765 was associated with the cell cycle and immune infiltration. Furthermore, we confirmed that the expression of ZNF765 had a strong connection with the infiltration level of various immune cells, such as B cells, CD4+ T cells, macrophages, and neutrophils. In addition, we found that ZNF765 was associated with m^6^A modification, which may affect the progression of HCC. Finally, drug sensitivity testing found that patients with HCC were sensitive to 20 drugs when they expressed high levels of ZNF765. In conclusion, ZNF765 may be a prognostic biomarker related to cell cycle, immune infiltration, m^6^A modification, and drug sensitivity for hepatocellular carcinoma.

## INTRODUCTION

Liver cancer is a worldwide disease, the fifth most common tumor, and the second most deadly cancer [1]. Hepatocellular carcinoma (HCC) accounts for the majority of primary liver cancers and has the characteristics of ease of transfer, high mortality, and a high recurrence rate [2], as well as a poor prognosis [3]. Most patients miss the optimal treatment time due to a late diagnosis. What we know is that HCC can be diagnosed by laboratory tests of serum biomarkers, including alpha-fetoprotein, and imaging techniques, including ultrasound, CT, and MRI imaging, and biopsy. Recently, advances have been made in the treatment of HCC [4, 5]. However, the survival and cure rates of patients with hepatocellular carcinoma are still not optimistic. Biomarkers, as biochemical indicators of changes in the structure or function of human organs, are often used to diagnose and stage diseases or to evaluate the usefulness of drugs and treatments [6]. Biomarkers of HCC that have been discovered for clinical use are mainly serum biomarkers such as alpha-fetoprotein. But these biomarkers are still not perfect for further efficient diagnosis and treatment. Exploring effective biomarkers for HCC has thus become the current task in order to improve the efficiency of diagnosis and optimize the treatment effect.

HCC is a complex ecosystem containing immune-related cells. The successful application of immune checkpoint inhibition in tumors has confirmed the important influence of the tumor microenvironment on tumor development [7]. Approximately 30% of early-stage HCCs have genomic evidence of immune activation, while 25% have no immune infiltration [8]. Understanding the interaction between cancer cells and their microenvironment is essential for developing new therapies and identifying biomarkers.

As the largest transcription factor family in the human genome, the ZNF family is widely involved in various biological processes in the human body. As the largest transcription factor family, zinc finger (ZNF) transcription factors are characterized by finger-like DNA domains, which require one or more zinc ions to stabilize the structure. At the moment, there is growing evidence that ZNF plays a significant role in HCC, ultimately regulating cell proliferation, apoptosis, invasion, and metastasis by adjusting the transcription of downstream target genes at a variety of regulatory levels. It is well on its way to becoming a new tumor biomarker and a therapeutic target in the treatment of HCC. It has been proven that some ZNF proteins have an effect on HCC. For example, ZNF384 can boost the proliferation of cancer cells [9], ZNF-148 can induce apoptosis of HCC cells and have a tumor suppressor effect [10], ZNF32 can escape apoptosis [11], and ZNF687 can induce HCC recurrence by adjusting hematopoietic cells in their proliferation and differentiation [12, 13], and so on. Considering that the ZNF family has such a powerful role in the occurrence and development of HCC, we selected ZNF765 (Zinc Finger Protein 765), an unexplored object, to be the object of study, trying to explore whether it can become an independent prognostic biomarker of HCC.

ZNF765 is a protein-coding gene in the ZNF family that is found on human 19q13.42. However, its role in cancer is rarely reported. One article correlated with cancer mentioned that ZNF765 was closely related to the regulation of the blood-tumor barrier. A significant mutation of ZNF765 has been found in chromophobe renal cell carcinoma, suggesting its potential role in kidney cancer [14, 15]. The mechanism of ZNF765 in HCC has not been reported, and its relationship with the prognosis is still unclear. In our research, it was found that ZNF765 can affect the development of HCC by affecting immune cells. Thus, we started from this point, trying to prove that ZNF765 can serve as a biomarker for HCC.

**Figure 1:**
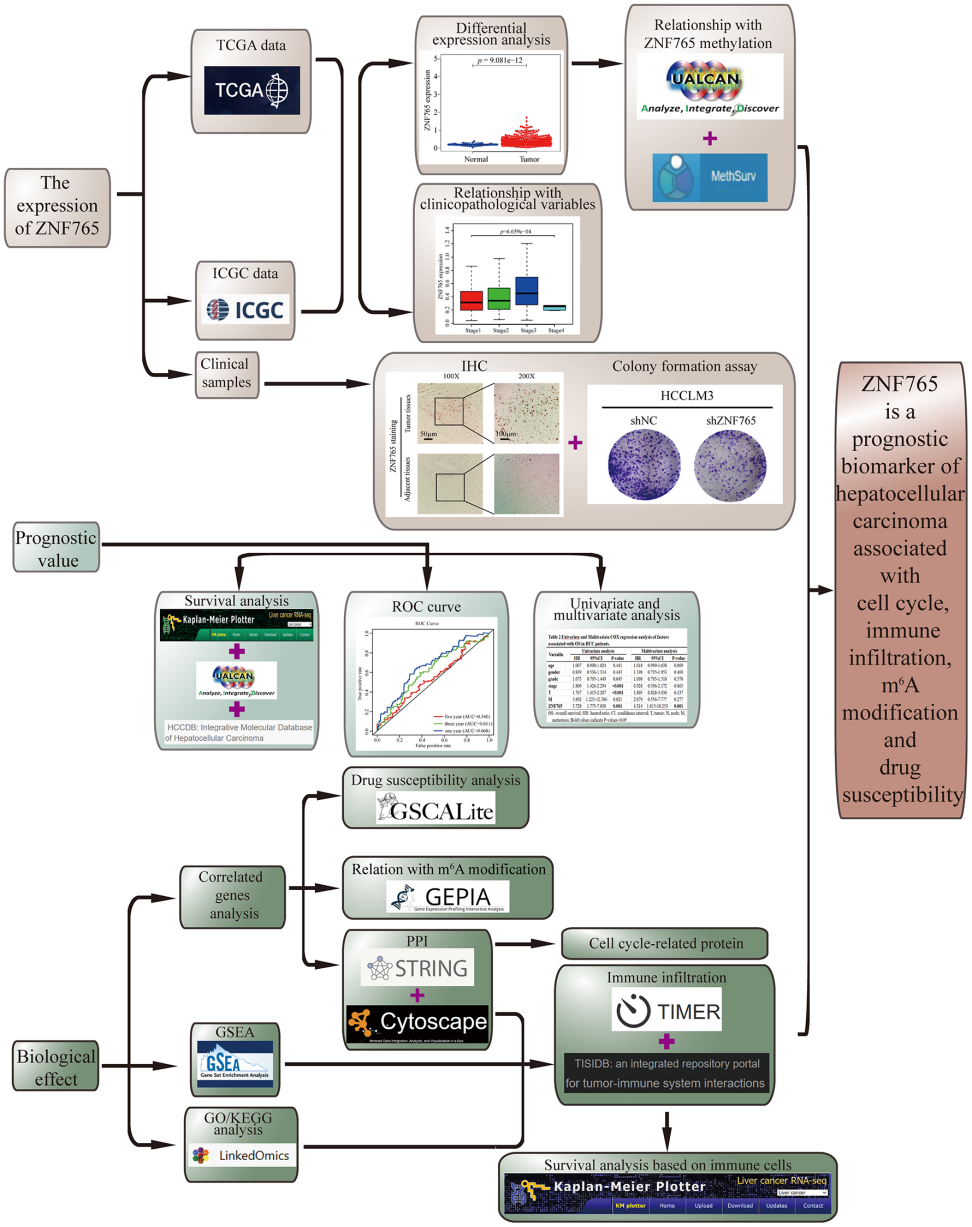
**Study workflow.** ZNF765 is a prognostic biomarker of hepatocellular carcinoma associated with cell cycle, immune infiltration, m6A modification, and drug susceptibility.

We used integrated bioinformatics methods, functional analysis, and some experimental verification in our article (Figure 1). We evaluated the expression of ZNF765 in HCC through a variety of public databases and clinical samples. The Wilcoxon rank sum test was exploited to study the relation between ZNF765 expression and clinicopathological characteristics in HCC. The influence of ZNF765 expression on HCC prognosis was examined by the Kaplan-Meier method. We investigated the transcriptome alterations and functional networks relevant to ZNF765 in HCC. The effect of ZNF765 expression on tumor immune infiltration is also in the process of being analyzed. The drug sensitivity test for ZNF765 is also covered. Our work revealed that ZNF765 is a new biomarker of HCC that can contribute to prognosis and treatment.

## RESULTS

### ZNF765 is upregulated in human HCC tissues

**Figure 2:**
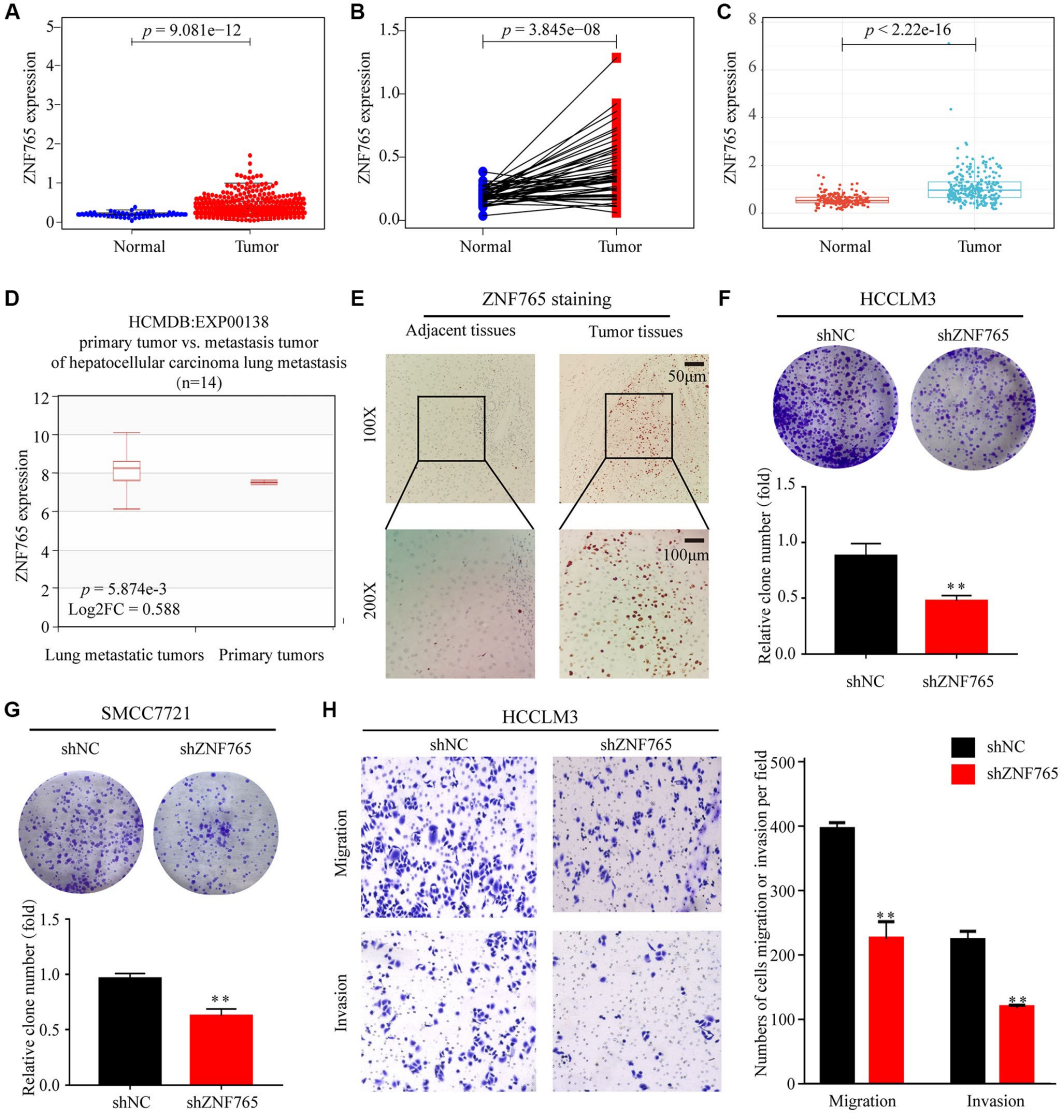
**The expression of ZNF765 in HCC and other cancers.** (**A**) ZNF765 mRNA levels in tumor and normal tissues based on the TCGA database (*p* = 9.061e-12). (**B**) Paired differential expression map of ZNF765 between 72 pairs of HCC tissues and normal tissues based on the TCGA database (*p* = 3.845e-08). (**C**) The mRNA expression level of ZNF765 in tumor and normal tissues in the ICGC (*p* = 2.22e-16). (**D**) HCMDB analysis of aberrant expression of ZNF765 in HCC patients. (**E**) Typical images of immunohistochemistry (IHC) in 40 pairs of HCC tissues showing the protein expression of ZNF765 in HCC and adjacent nontumor tissues. Colonies formed by HCCLM3 (**F**) and SMCC7721 (**G**) cells transfected with control shRNA or shRNA targeting ZNF765. The panels are a quantification of the results of the colony formation assay (^**^p < 0.01). (**H**) Representative data from Transwell migration and invasion assays performed with the ZNF765 knock-down cells.

To dig out ZNF765 expressions in various cancerous and normal tissues, some of the works listed below were performed. First, our study analyzed the TCGA-RNA sequence data in TIMER. It proclaimed that, in comparison with normal tissues, ZNF765 had an increased expression in a wide range of cancer tissues. It was also true in LIHC (Supplementary Figure 1). Then, the Wilcoxon rank sum test was used to generate differential expression maps and paired differential expression maps to examine the specific circumstances of ZNF765 expression between normal and tumor samples (Figure 2A, 2B). What’s more, we used ICGC, another database, to conduct the same analysis (Figure 2C) and got a similar outcome to our preliminary result in TCGA. To sum up, in the whole transcriptome sequencing (RNA-seq) dataset, ZNF765 was clearly overexpressed in tumor tissues. Moreover, we used the HCMDB online website to draw the expression box plots of ZNF765 in primary tumor and lung metastatic tumor tissues. As a result, ZNF765 was found to be significantly more abundant in metastatic cancer than in primary cancer (Figure 2D). Furthermore, analysis of 10 HCC cohorts in the HCCDB database clearly showed that ZNF765 mRNA expression in HCC tissues was obviously higher than that in other tissues (Table 1). Besides, we used clinical samples to verify our preliminary results. IHC was adopted to test the protein content of ZNF765, and its results indicated that the protein level of ZNF765 was elevated in 40 pairs of HCC tissues (Figure 2E). In the colony formation assay, decreased expression of ZNF765 resulted in a reduced colony-forming capacity of HCC cells (Figure 2F, 2G). In the Transwell Assay, ZNF765 knockdown significantly reduced the invasion and migration abilities of HCCLM3 cells (Figure 2H). Anyway, our results proved the overexpression of ZNF765 in HCC tissues, which may be related to HCC progression.

**Table 1:** Analysis of mRNA expression of ZRANB1 in the HCCDB database.

**Dataset**	***p* value**	**Type**	**Nums**	**Mean**	**STD**	**IQR**
HCCDB1	**9.55E-08**	HCC	100	6.199	0.6365	0.9087
		Adjacent	97	5.794	0.3365	0.3778
HCCDB3	**1.32E-18**	HCC	268	0.1659	0.09024	0.084
		Adjacent	243	0.1112	0.03066	0.038
		Cirrhotic	40	0.1485	0.04295	0.07175
		Healthy	6	0.1038	0.01545	0.006
HCCDB4	**0.0005271**	HCC	240	6.639	0.1155	0.1243
		Adjacent	193	6.602	0.1046	0.1279
HCCDB11	**0.02634**	HCC	88	6.748	0.7977	1.139
		Adjacent	48	7.083	0.8452	1.146
HCCDB12	0.2862	HCC	81	5.348	0.9213	1.023
		Adjacent	80	5.477	0.5696	0.4129
HCCDB13	**0.000521**	HCC	228	4.141	0.1739	0.2127
		Adjacent	168	4.087	0.1357	0.1683
HCCDB15	**0.0006856**	HCC	351	6.713	0.8377	1
		Adjacent	49	6.436	0.4569	0.42
HCCDB16	0.4082	HCC	60	9.822	0.7276	0.6234
		Adjacent	60	9.926	0.6489	0.5297
HCCDB17	**0.0000512**	HCC	115	7.049	0.1159	0.1325
		Adjacent	52	7.141	0.1356	0.185
HCCDB18	**2.47E-27**	HCC	212	0.9681	0.3645	0.48
		Adjacent	177	0.6241	0.1942	0.2

Abbreviations: STD: standard deviation; IQR: Inter Quartile Range. Bold values indicate *p* values < 0.05.

### Relationship between ZNF765 expression and clinicopathological variables in HCC

**Figure 3:**
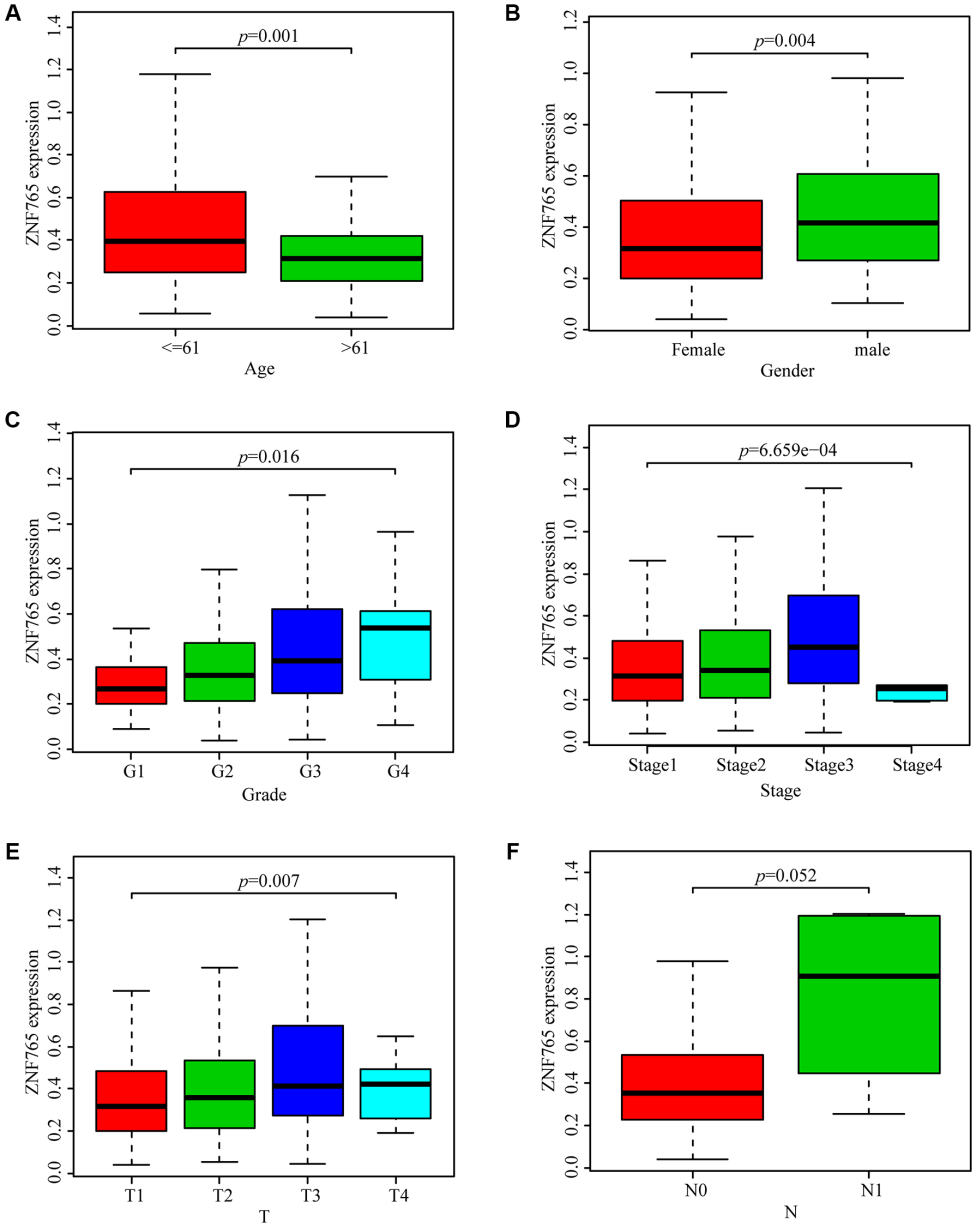
**Box plots exploring the relationship between ZNF765 expression and clinicopathological characteristics.** (**A**) Age; (**B**) Gender; (**C**) Grade; (**D**) Stage; (**E**) T (size of the tumor); (**F**) N (lymph node metastasis).

With the aim of investigating the relationship between ZNF765 expression and HCC sufferers’ clinicopathological features based on the TCGA database, we selected relevant data for analysis and produced a series of related box-plots using R software. We found that the expression of ZNF765 was higher in the ≤ 61 group (Figure 3A). And the expression of ZNF765 in the two genders was different (Figure 3B). In addition, the expression of ZNF765 increased with the grade (Figure 3C). Simultaneously, we found that the expression of ZNF765 was also high in stages 1, 2, and 3 (Figure 3D). Besides, ZNF765 expression was associated with tumor size (Figure 3E). However, the expression of ZNF765 was not correlated with N (lymph node metastasis) (Figure 3F). For the purpose of analyzing the relationship between ZNF765 expression and poor clinicopathologic variables, we further adopted logistic regression. And the consequences suggested that high ZNF765 expression was notably related to age (OR = 0.55 for >60 vs. 60), gender (OR = 1.78 for female vs. male), grade (OR = 4.03 for III vs. I), stage (OR = 1.75 for III vs. I), and T (OR = 1.37 for T2 vs. T1), but not related to N (OR = 3.05 for N1 vs. N0) (Table 2). The above results indicated that ZNF765 expression was closely correlated with clinicopathological characteristics.

**Table 2:** Logistic analysis of the association between ZNF765 expression and clinical characteristics.

**Clinical characteristics**	**Total (*N*)**	**Odds ratio in ZNF765 expression**	***p* value**
Age (>60 vs. ≤60)	376	0.55 (0.37–0.84)	**0.005**
Gender (Female vs. Male)	377	1.78 (1.15–2.78)	**0.01**
Grade (III vs. I)	372	4.03 (2.06–8.18)	**<0.001**
Stage (III vs. I)	353	1.75 (1.03–2.98)	**0.038**
T (T2 vs. T1)	374	1.37 (0.83–2.27)	0.215
N (N1 vs. N0)	261	3.05 (0.38–62.07)	0.337

Abbreviations: T: tumor; N: node; M: metastasis; Bold values indicate *p* values < 0.05.

### ZNF765 expression is an independent prognostic factor that is associated with poor prognosis in HCC patients

**Figure 4:**
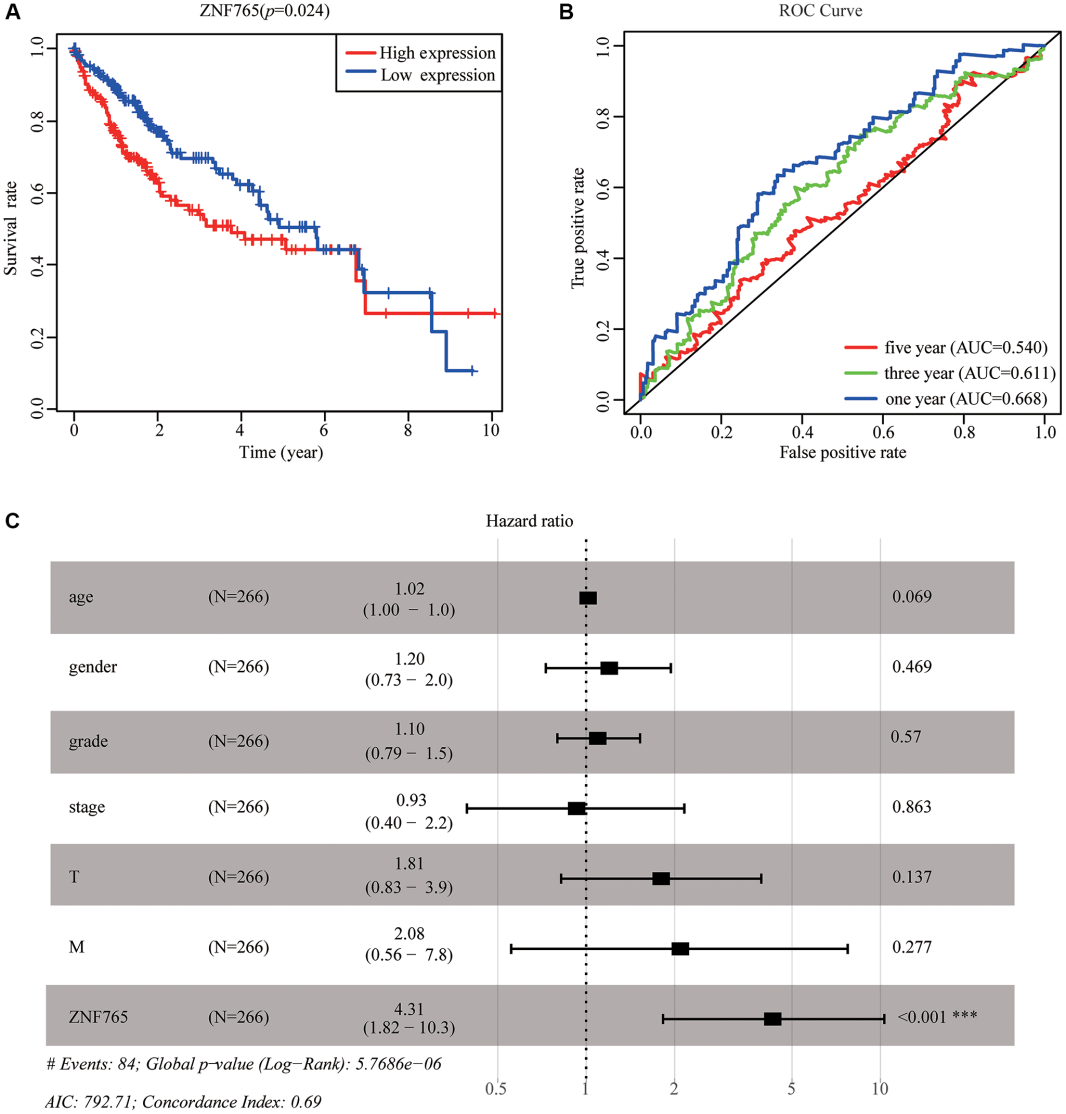
**The effectiveness of ZNF765 in predicting prognosis.** (**A**) HCC patients with a lower expression level of ZNF765 had favorable a prognosis (*p* = 0.024). (**B**) ROC curves for the 1-, 3-, and 5-year survival according to the expression level of ZNF765. Abbreviation: AUC: the area under the curve; ROC: receiver operating characteristic. (**C**) A forest plot of the results of the multivariate analysis. ^*^p < 0.05; ^**^p < 0.01; ^***^p < 0.001. Abbreviations: HR: hazard ratio; CI: confidence interval; T: tumor; N: node, M: metastasis; OS: overall survival; AIC: Akaike’s information criterion.

We adopted TCGA-LIHC data to explore how overexpressed ZNF765 influences the prognosis of HCC patients. According to the results, we found that high expression of ZNF765 could result in a poor prognosis (Figure 4A). Designed to dive into the prognostic value of ZNF765 expression in HCC, Kaplan-Meier Plotter tools were applied to detect the prognosis of ZNF765. The Kaplan-Meier curve and log rank test analysis revealed that the increased ZNF765 level was significantly related to overall survival (OS), relapse-free survival (RFS), progression-free survival (PFS), and disease-specific survival (DSS) (Supplementary Figure 2A–2D) (*p* < 0.05). The same analysis on the HCCDB website also presented a similar result (Supplementary Figure 2E). High mRNA levels of ZNF765 were forecast to have low OS, RFS, PFS, and DSS. The area under the ROC curve was used to assess the sensitivity and specificity of ZNF765 expression in predicting survival outcomes (Figure 4B). The results displayed that the area under the ROC curve (AUC) for 1-, 3-, and 5-years was 0.668, 0.611, and 0.540, respectively. Univariate and multivariate analysis using the Cox regression model illustrated that ZNF765 expression is an independent prognostic factor for the survival of HCC patients (Supplementary Table 1). Additionally, the forest plot also consistently showed the equivalent result (Figure 4C). Besides, we used the UALCAN website to make an analysis among expressions of ZNF765, some clinicopathological features, and survival probability. And we found that when clinicopathological features stayed the same, high expression of ZNF765 always led to a poor survival probability (Supplementary Figure 2F–2H). In conclusion, ZNF765 was an independent prognostic factor for HCC.

### The relationship between ZNF765 methylation and its expression

**Figure 5:**
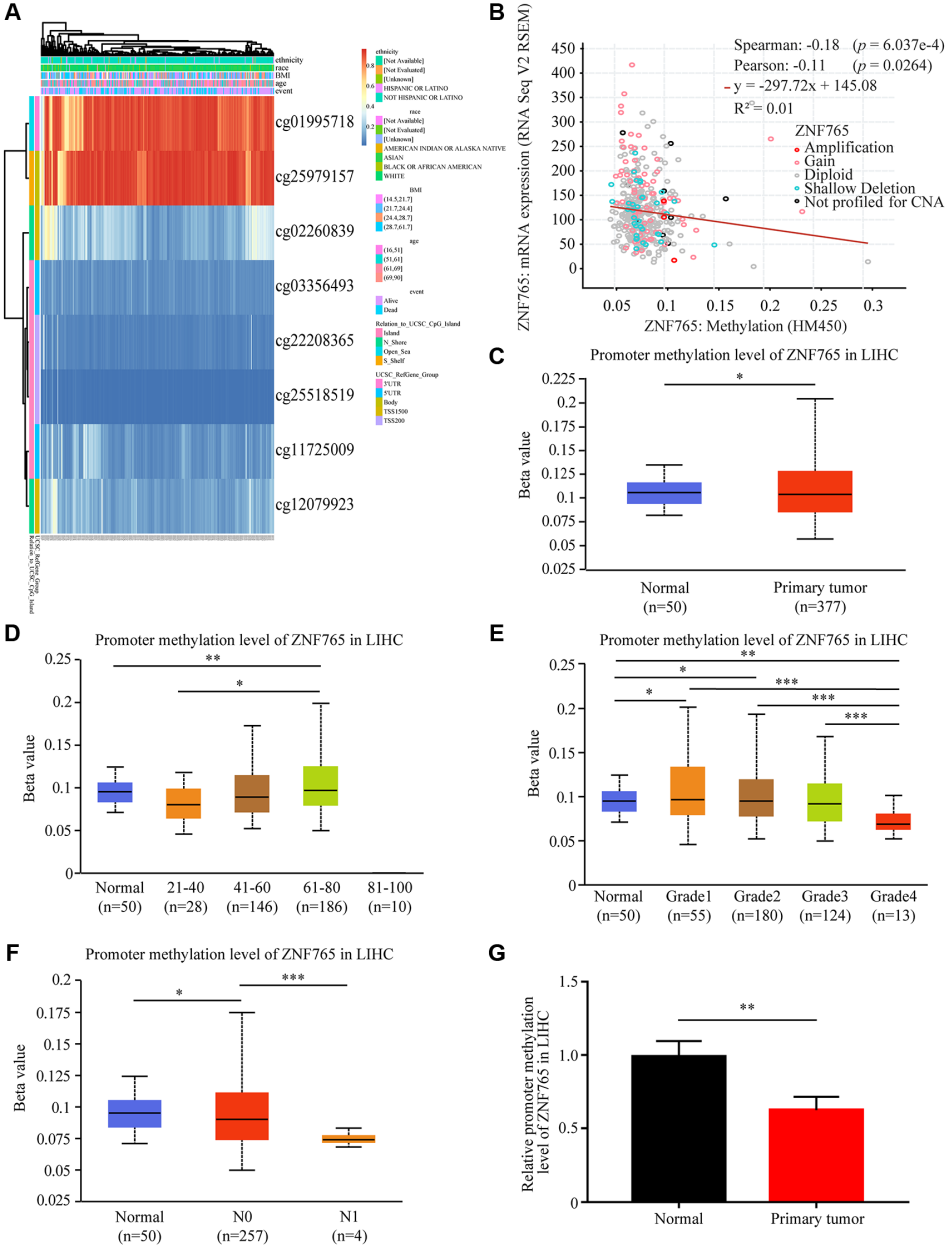
**DNA methylation of ZNF765 in HCC.** (**A**) Heatmap for ZNF765 in HCC. (**B**) The correlation between ZNF765 methylation and its expression level (*n* = 521). (**C**) A boxplot of ZNF765 promoter methylation levels in normal and HCC samples. (**D**) Boxplot demonstrating the relative promoter methylation level of ZNF765 in healthy people of any age or HCC patients aged 21–40, 41–60, 61–80, or 81–100 years. (**E**) A boxplot depicting the relative promoter methylation level of ZNF765 in healthy people and HCC patients of different genders. (**F**) Boxplot showing the relative promoter methylation level of ZNF765 in normal individuals or HCC patients in grades 1, 2, 3, or 4. (**G**) Boxplot depicting the relative promoter methylation level of ZNF765 in normal individuals with any nodal metastasis status or HCC patients with N0 and N1 nodal metastasis. ^***^p < 0.001.

DNA methylation, an essential process in the epigenetic modification of the genome, has something to do with the disease process [16]. Methylation, in particular, has the potential to cause genome instability and stimulate correlated genes. We attempted to investigate whether ZNF765 DNA methylation could affect its expression in LIHC and elucidate the relationship between the two. Through the MethSurv web platform, we made a DNA methylation heatmap of ZNF765 that is shown in Figure 5A. Most sites are hypomethylated (Figure 5A). Then, we further explored the connection between ZNF765 methylation and gene expression with cBioPortal, and the result indicated that gene methylation negatively correlated with ZNF765 expression (Spearman = -0.18, *p* = 6.037e-4; Pearson = −0.11, *p* = 0.0264) (Figure 5B). The methylation levels of ZNF765 in LIHC samples with different clinicopathological classifications were analyzed by UALCAN. The DNA methylation level of ZNF765 in tumor samples from LIHC was slightly lower than that of normal samples (*p* < 0.001; Figure 5C). When it came to age, the methylation level decreased in the 41-60 and 61-80 groups (Figure 5D). In addition, methylation levels of ZNF765 decreased gradually as their grade or nodal metastasis status increased (Figure 5E, 5F). Besides, the ZNF765 promoter methylation level was also detected, which showed that the methylation level of the ZNF765 promoter in LIHC tissues was lower than that in normal tissues (Figure 5G). Therefore, the decreased methylation level of ZNF765 may lead to an increase in the expression level of ZNF765.

### Pathway enrichment analysis of ZNF765 in HCC

To understand the biological importance of ZNF765 in HCC in depth, we applied the function module of LinkedOmics. We did enrichment analyses on the website. The results of functional enrichment and GO analysis indicated that ZNF765 was functionally related to the cell cycle and DNA replication. Some enrichment terms can prove this conclusion, including chromosome segregation, DNA replication, cell cycle checkpoint, G0 to G1 transition, cell cycle G2/M phase transition, and meiotic cell cycle (Supplementary Figure 3A). Besides, KEGG pathway analysis showed that there was an enrichment of genes extremely related to the cell cycle (Supplementary Figure 3B). For further investigation on the biological meaning of ZNF765 in HCC, we employed GSEA to analyze the datasets based on the TCGA LIHC with different expression groups of ZNF765. 139 of 178 pathways were up-regulated, and 81 of them met the conditions of NOM *p* < 0.05, FDR < 0.05, and NES > 1.7. The pathways associated with promoting cell adhesion and tumorigenesis in the ZNF765 overexpression group included FOCAL adhesion, the pathway in cancer, the MAPK signaling pathway, and the P53 signaling pathway. The immune infiltration pathways included B cell receptor signaling, T cell receptor signaling, leukocyte transendothelial migration, FC-γ-R-mediated phagocytosis, and the TGF-β signaling pathway. Cell cycle terms included cell cycle, DNA replication, and RNA degradation. The enrichment results are summarized in Supplementary Figure 3C.

### ZNF765 Co-expression networks in HCC

**Figure 6:**
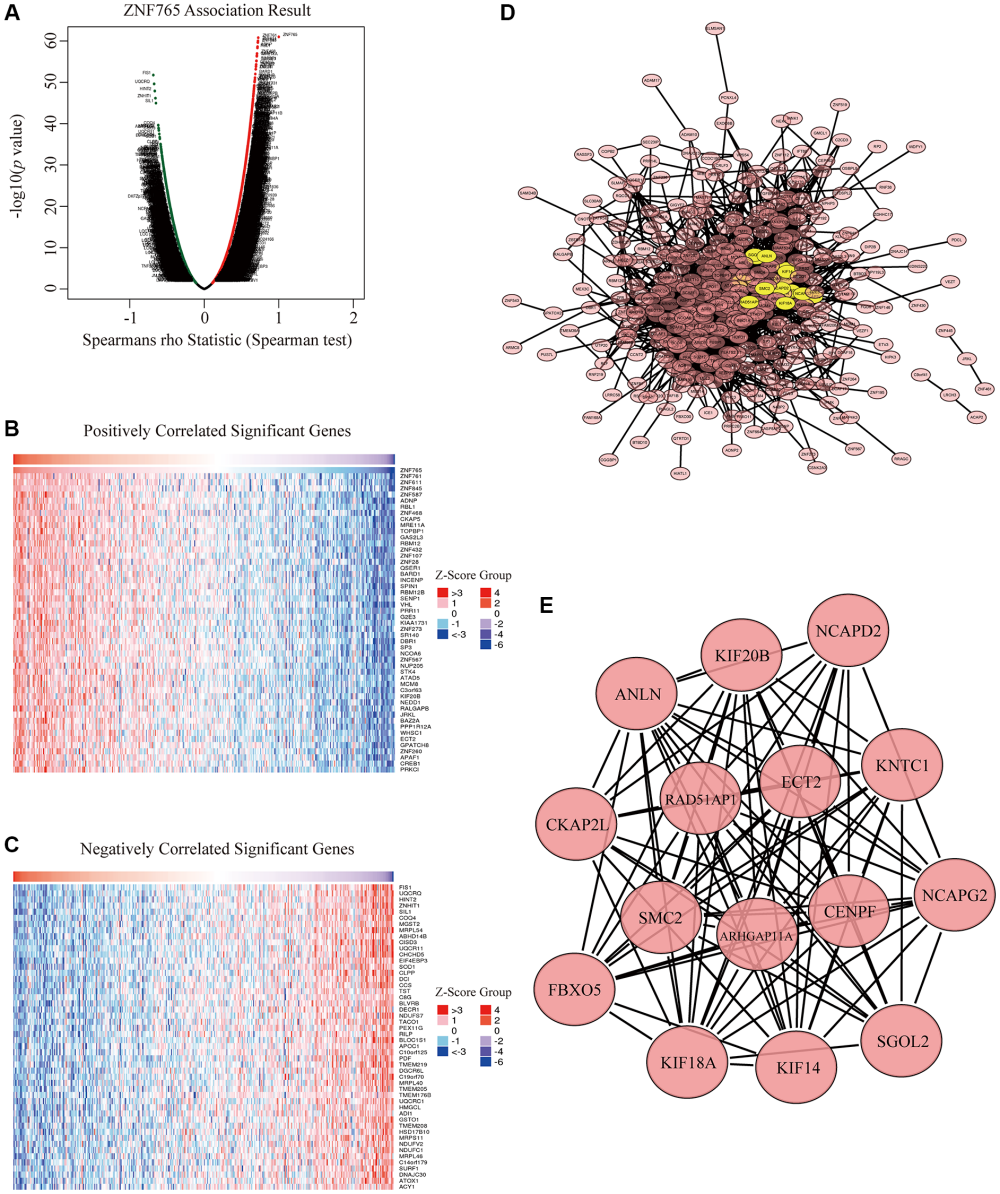
**Co-expression genes and protein-protein interaction (PPI) network of ZNF765 in HCC.** (**A**) A correlation analysis was used to assess correlations between ZNF765 and genes differentially expressed in HCC. Red shows positively correlated genes, and green indicates negatively correlated genes. False discovery rate, FDR < 0.0. (**B**, **C**) Heat maps show genes positively and negatively correlated with ZNF765 in HCC (Top 50). (**D**) The most significant module selected by the MCODE plugin (degree cut-off = 2, node score cut-off = 0.2, k-core = 2, and max. Depth = 100). (**E**) Hub genes.

To better understand the biological effects of ZNF765 in HCC in depth, we applied LinkedOmics, aiming to test the ZNF765 co-expression genes in HCC. There are 13490 genes that have a significant positive correlation with ZNF765, and they are represented by dark red dots. Meanwhile, 6432 genes that negatively correlated with ZNF765 were represented by dark green dots (Figure 6A). In heatmaps (Figure 6B, 6C), 50 notable gene sets with observably positive and negative correlations with ZNF765 were marked and listed. Then, to dig into the functions of ZNF765, we further selected the top 500 co-expressed genes among genes in the Volcano Plot based on Spearman’s coefficient, and then we analyzed these genes with the STRING database and visualized the result with Cytoscape (Figure 6D). Then we chose these co-expressed genes to do our significant part, the PPI network, using Cytoscape (the MCODE plug-in) (Figure 6E). From the above results, we could tell that the module with the highest score consisted of 15 genes. These 15 genes were regarded as the hub genes because they were marked in yellow. Since ARHGAP11A, ECT2, and ANLN are known to be closely correlated to cell growth or the cell cycle, based on the above analysis results, we inferred that the effect of ZNF765 on the survival of patients with hepatocellular carcinoma may be relevant to the cell cycle.

### ZNF765 is correlated with tumor purity, immune infiltration and escape in HCC

**Figure 7:**
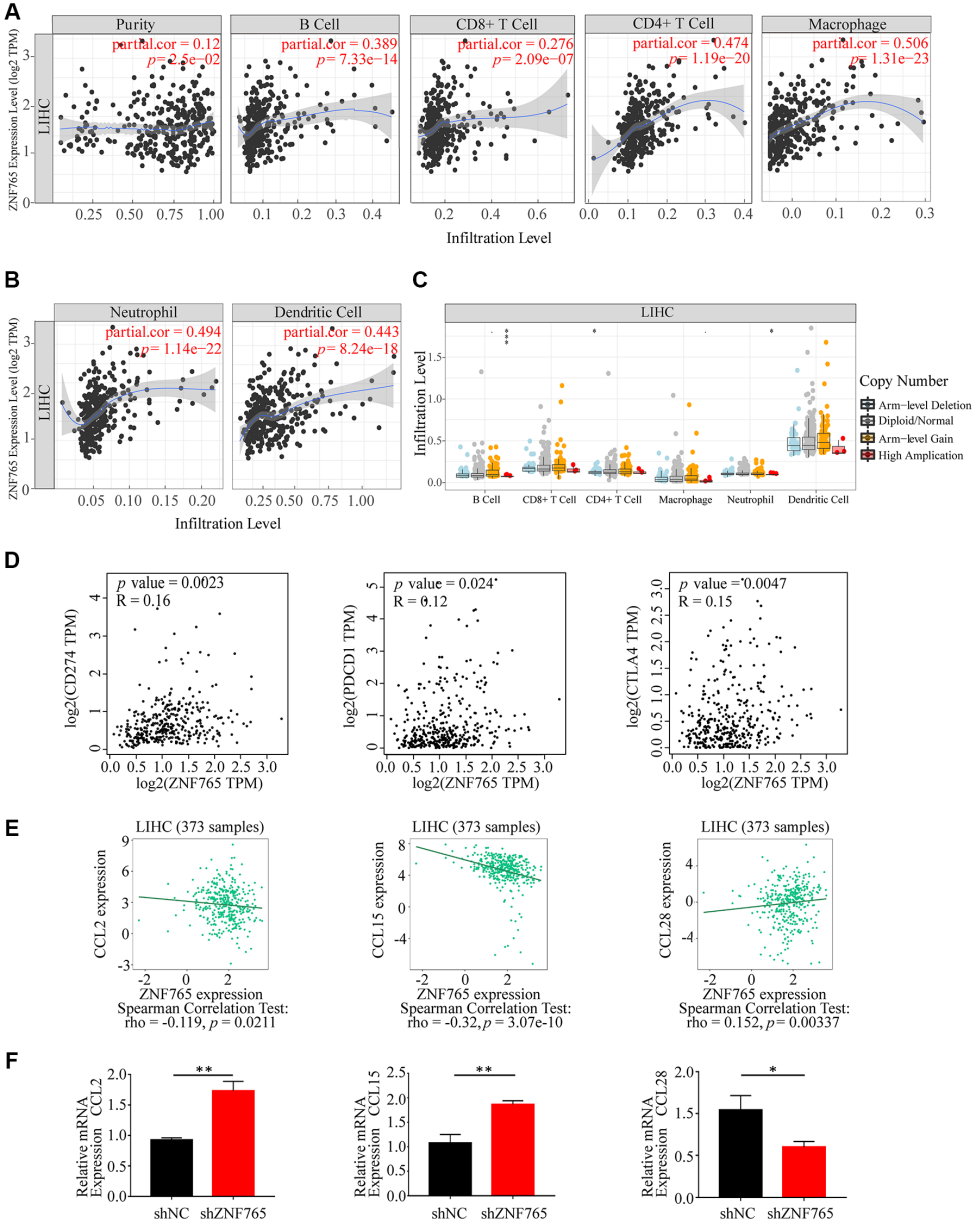
**Correlations of ZNF765 expression with immune infiltration level and LIHC-related chemokines.** (**A**, **B**) ZNF765 expression is negatively related to tumor purity, infiltrating levels of B cells, CD8+ T cells, CD4+ T cells, macrophages, neutrophils, and dendritic cells in HCC. (**C**) ZNF765 CNV affects the infiltrating levels of B cells, CD4+ T cells, and neutrophil cells in HCC (^*^p < 0.05, ^***^p < 0.001). (**D**) The mRNA correlation between CD274/PDCD1/CTLA4 and ZNF765 in the LIHC data of TCGA. (**E**) The association between ZNF765 and LIHC-related chemokines. (**F**) CCL2/CCL15/CCL28 relative mRNA expression in shNC and shZNF765.

Tumor occurrence and development were linked to the number of immune cells infiltrated [17]. To determine whether ZNF765 expression is correlated with immune cell infiltration, TIMER was adopted to evaluate the relevance between the two. Our outcomes indicated that ZNF765 expression showed a slightly positive connection with the purity of HCC tumors (r = 0.12, *p* = 2.5e-02) and was positively correlated with the infiltration levels of B cells (r = 0.389, *p* = 7.33e-14), CD8+ T cells (r = 0.276, *p* = 2.09e-07), CD4+ T cells (r = 0.474, *p* = 1.19e-20), macrophages (r = 0.506, *p* = 1.31e-23), neutrophils (r = 0.494, *p* = 1.14e-22), and dendritic cells (r = 0.443, *p* = 8.24e-18). Further observation, ZNF765 expression in HCC is significantly correlated with the infiltrating levels of macrophages, CD4+ T cells, and neutrophils (Figure 7A, 7B). Aiming to go deep into the correlation between ZNF765 and various immune cells, the relationship between ZNF765 mRNA expression level and immune marker genes was evaluated via the TIMER database. The result of the analysis indicated that ZNF765 was positively associated with some specific immune cell gene markers of B cells, T (general) cells, CD8+ T cells, monocytes, TAM, M1 and M2 macrophages, neutrophils, natural killer cells, and dendritic cells (Supplementary Figure 4). The specific quantitative results can be known from Supplementary Table 2.

We further compared the abundance distribution in tumor-infiltrating immune cells with different ZNF765 somatic copy number alteration (SCNA) via TIMER. B cells were the primary infiltrating immune cells of the ZNF765 gene in HCC with high amplification (all *p* value < 0.05) (Figure 7C). Meanwhile, our study also explored the correlation between ZNF765 expression and well-known T cell checkpoints (such as PD-1, PD-L1, and CTLA-4) in the GEPIA database. ZNF765 expression and the expression of PD-1, PD-L1, and CTLA-4 were found to have significant relationships in HCC (Figure 7D), implying that ZNF765 may be involved in immune escape. In addition, through the TISIDB website, we found that HCC-related chemokines such as CCL2 and CCL15 were negatively correlated with ZNF765, while CCL28 was positively correlated (Figure 7E). We experimentally verified the expression of immune-related chemokines in ZNF765-silenced HCC cells (Figure 7F). Compared with shNC, the expression of CCL2 and CCL15 was increased and the expression of CCL28 was significantly decreased in ZNF765-silenced cells. Therefore, we hypothesized that the correlation between ZNF765 and immune infiltration may be due to chemokines. In a word, ZNF765 had a positive relation with the purity of the tumor, and the expression ZNF765 was obviously related to the level of HCC immune infiltration and evasion.

### Prognostic analysis of ZNF765 expression in HCC based on immune cells

**Figure 8:**
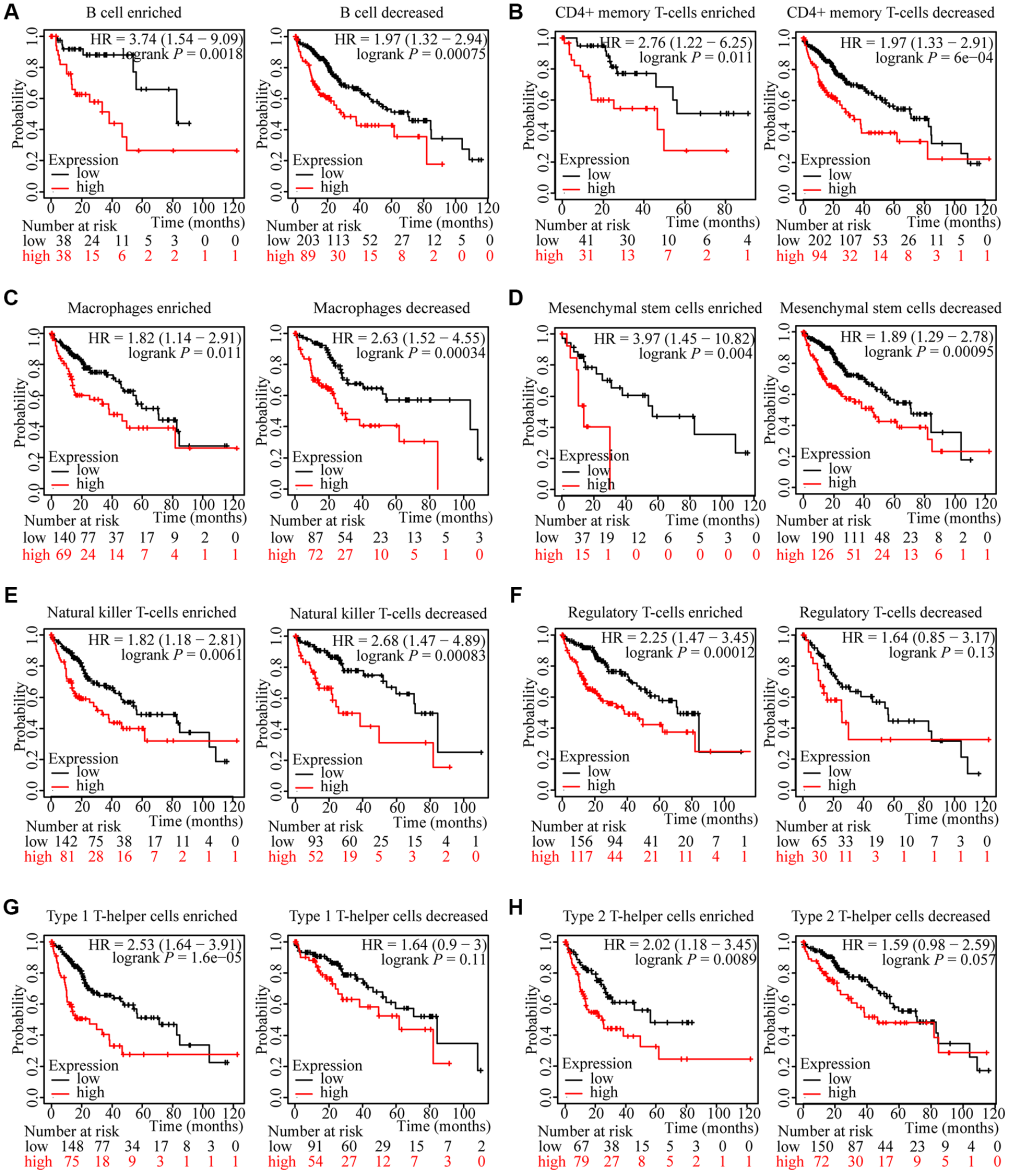
**Kaplan-Meier survival curves according to high and low expression of ZNF765 in immune cell subgroups in HCC.** (**A**–**H**) Relationships between ZNF765 of different immune cell subgroups and prognoses in HCC.

We have attested that ZNF765 expression was associated with immune infiltration in hepatocellular carcinoma and with poor prognosis in patients. We attempted to investigate whether ZNF765 affects the prognosis of HCC patients through immune cells. We analyzed the prognosis of HCC patients according to different immune cell types and their different enrichment levels using Kaplan-Meier Plotter tools. The results of grouping analysis according to the number of immune cells showed that the survival of HCC patients was similar under the conditions of enrichment and non-enrichment of various immune cells like B cells, CD4+ memory T cells, macrophages, and so on (Figure 8A–8E). Interestingly, the degree of infiltration of certain kinds of immune cells can affect the prognosis of HCC patients. For example, high expression of ZNF765 in the enriched regulatory T-cells, enriched Type 1 T-helper cells, and enriched Type 2 T-helper cell cohorts in HCC was linked to poor prognosis. In the decreased regulatory T-cell, decreased Type 1 T-helper cell, and decreased Type 2 T-helper cell cohorts, however, there was no association of ZNF765 levels with HCC patient prognosis (Figure 8F–8H). These findings suggested that the degree of infiltration of multiple immune cell subtypes, especially T cell subtypes, has a certain influence on the prognosis of HCC patients. Through TIMER, we also found that the expression of ZNF765 was significantly correlated with gene markers of different types of T cells (Supplementary Table 3). In conclusion, we speculated that ZNF765 may affect the prognosis of HCC patients through T-cell infiltration.

### Corrections of ZNF765 expression with m^6^A modification in HCC

**Figure 9:**
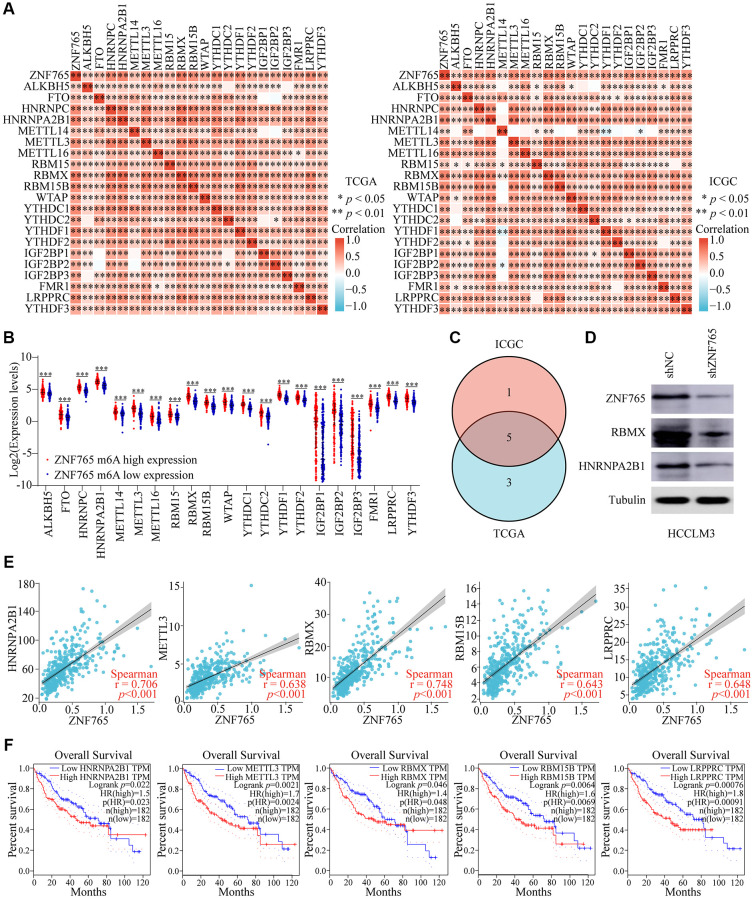
**Corrections of ZNF765 expression with m^6^A modification in HCC.** (**A**) The correlation between ZNF765 expression and the expression of m6A-modified genes was investigated by the Spearman statistical method using the TCGA and ICGC databases. (**B**) Distinct m6A-related gene expression in HCC patients with different expressions of ZNF765. (**C**) Five genes were found at the intersection of the TCGA and ICGC databases. (**D**) A Western blot was used to detect ZNF765, RBMX, and HNRNPA2B1 protein expression in HCCLM3 cells stably transfected with the control shRNA or the ZNF765 shRNA. (**E**) The correlation between ZNF765 and m^6^A-modified genes was analyzed by the scatter plot. (**F**) The overall survival of HCC patients was separated into two groups based on the high and low expression of these five m^6^A genes. ^*^p < 0.05; ^**^p < 0.01; ^***^p < 0.001.

N6-methyladenosine (m^6^A), a kind of internal modification that occurs at the N6-position of adenosine, most frequently happens in eukaryotic mRNA [18]. Numerous studies have found that m^6^A RNA methylation can play a role in the onset and progression of various diseases, particularly cancers, by influencing RNA metabolism [19]. Therefore, we attempted to investigate whether there was a correlation between ZNF765 and 21 m^6^A-related genes in HCC using the TCGA and ICGC databases, and our research findings indicated that there was a close relationship between the two (Figure 9A). Meanwhile, m^6^A-related gene expression was also different in HCC patients with different expressions of ZNF765 (Figure 9B). Next, we used Venn diagram analysis to obtain five genes with correlation coefficients greater than 0.6 with ZNF765 in the two databases, namely HNRNPA2B1, METTL3, RBMX, RBM15B, and LRPPRC (Figure 9C). Western blotting verified the inhibitory effect of ZNF765 knockdown on the expression of HNRNPA2B1 and RBMX proteins with a correlation greater than 0.7 (Figure 9D). Its correlation with ZNF765 was shown again in the scatter plot (Figure 9E). Further, overexpression of these m^6^A-related genes was found to predict lower survival of HCC patients in GEPIA (Figure 9F). Furthermore, the results of risk score and survival analysis showed that HNRNPA2B1, METTL3, and LRPPRC were risk factors for HCC when ZNF765 was highly expressed (Supplementary Figure 5A, 5B). However, HNRNPA2B1, METTL3, and LRPPRC had no effect on the prognosis of HCC patients when ZNF765 was lowly expressed (Supplementary Figure 5C). These results suggested that ZNF765 may affect patients with HCC through m^6^A-related regulatory factors.

### Drug susceptibility analysis associated with ZNF765

**Figure 10:**
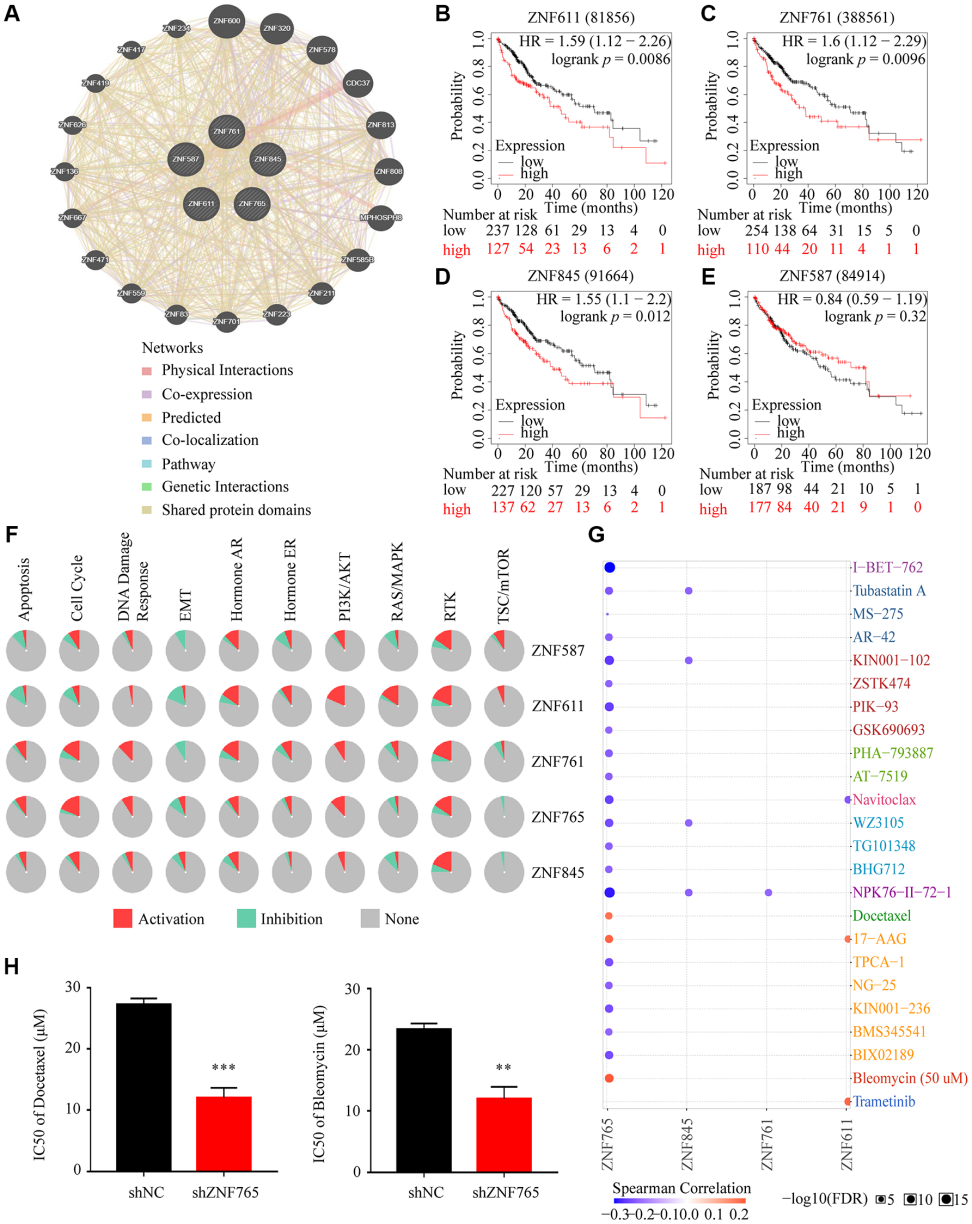
**Drug susceptibility analysis associated with ZNF765.** (**A**) The correlation between ZNF765 and the four jointly expressed genes most associated with it was explored by GeneMANIA analysis. (**B**–**E**) Took advantage of the Kaplan-Meier plotter to analyze the relationship between the expression of these four genes and the overall survival of HCC patients. (**F**) Pathway analyses were studied by the GSCA Lite website. (**G**) We used the GSCA Lite website to display drug susceptibility with these five hub genes. (**H**) HCCLM3 cells transfected with the shNC or shZNF765 were treated with Docetaxel and Bleomycin for 72 h, and then, cell viability was measured. ^*^p < 0.05; ^**^p < 0.01; ^***^p < 0.001.

Tumors being resistant to the drug may be the chief cause of the poor effects of chemotherapy in hepatocellular carcinoma [20]. Consequently, we primarily identified the first four jointly expressed genes most associated with ZNF765 from the volcanic map (Figure 6A) to conduct drug susceptibility analysis, namely ZNF587, ZNF611, ZNF761, and ZNF845. The GeneMANIA analysis revealed that they are very close relatives (Figure 10A). Additionally, the Kaplan-Meier plotter was used to determine whether the expression of these four genes affected the overall survival of HCC patients. In general, the results showed that when these four genes expressed at elevated levels, poor overall survival can occur (Figure 10B–10E). Pathway enrichment analysis through the GSCALite website revealed that the expression of these five genes, in particular, promoted the RTK pathway. And the high expression of ZNF765 activated the cell cycle, PI3K/AKT, RTK, hormone ER, apoptosis pathways, and DNA damage response, while inhibiting TSC/mTOR, RAS/MAPK, and EMT pathways (Figure 10F). Additionally, the result of the drug susceptibility analysis illustrated that cells with high ZNF765 expression were sensitive to 20 drugs, while they were resistant to Docetaxel, 17-AAG, and Bleomycin (Figure 10G). Previous articles reported that docetaxel and bleomycin are safe and effective treatments for HCC [21, 22], and our study confirmed that the knockdown of ZNF765 will increase the sensitivity of hepatocellular carcinoma cells to these two drugs (Figure 10H), which may provide a reference for drug treatment in HCC patients.

## DISCUSSION

Hepatocellular carcinoma is a worldwide problem [23]. A significant number of HCC patients cannot receive timely diagnosis and treatment because of a lack of obvious symptoms and diagnostic markers in the early stage. Moreover, the percentage of advanced radical resectable HCC is low (10%-20%), and the prognosis is poor (total mortality to morbidity ratio of 0.95) [24, 25]. The rise of molecular-targeted therapies offers more hope for HCC patients. However, the further development of targeted drugs is currently almost at a standstill [26], making it necessary to identify more biomarkers for HCC patients. The objective of our study was to assess the expression, prognostic value, and biological effect of ZNF765 in hepatocellular carcinoma by using a variety of bioinformatics analysis methods.

First, we used the TCGA and ICGC databases to analyze the expression of ZNF765 in HCC and its prognostic effect. As a result, ZNF765 was found to be overexpressed in HCC patients and was correlated with clinicopathological features. With the continuous development of the tumor, the expression level gradually increased, and high expression predicted poor survival. Moreover, immunohistochemistry and a colony formation assay confirmed the overexpression of ZNF765 in hepatocellular carcinoma and its promoting effect on the proliferation of this cancer. The COX model showed that ZNF765 could be an independent prognostic factor for hepatocellular carcinoma, which highlighted the prognostic role of ZNF765 in hepatocellular carcinoma. The above results tentatively confirm the involvement of ZNF765 in the progression of hepatocellular carcinoma.

DNA methylation is a form of DNA modification that plays a major role in gene expression in mammalian genomes [27]. We hypothesized that a low promoter methylation level resulted in high ZNF765 expression and validated this hypothesis using UALCAN, MethSurv, and cBioPortal. Based on the results, we believe that hypomethylation led to high expression of ZNF765, which even affected the survival of patients.

Subsequently, the function of ZNF765 was further studied. Co-expressed genes were identified, and PPI analysis was performed. Some proteins interacting with ZNF765 were found to be involved in the cell cycle. At the same time, enrichment analysis also revealed cell cycle, DNA replication, and immune-related pathways. Uncontrolled tumor cell proliferation caused by abnormal activity of various cyclins is characteristic of cancer [28]. It is known that a variety of cell cycle proteins play a key role in hepatocellular carcinoma, such as Cyclin D1 (CCND1), C-myc or Ras, cyclin D2 (CCND2), etc. [29]. Among the co-expressed genes of ZNF765, Arhgap11a expresses itself in a cycle-dependent manner [30], and ECT2, as a guanine nucleotide exchange factor, plays an important role in cell division and cell cycle regulation [31]. In particular, ANLN may be involved in the pathway that mediates abnormal cell division in the cell cycle in HCC [32]. As a result, we hypothesized that ZNF765 might affect HCC by affecting the cell cycle.

The cellular component of the tumor microenvironment (TME) is highly complicated [33], and multiple immune cells are involved in cancer immune evasion and the immunotherapy response [34], as well as playing a key role in HCC initiation and progression [35]. In addition, the results of the above pathway enrichment analysis have suggested that ZNF765 may be involved in immune-related pathways. For these reasons, we attempted to further explore the potential association between ZNF765 and the immune microenvironment. Studies have shown that the interaction between HCC cells and macrophages can facilitate the proliferation and metastasis of cancer cells through the up-regulation of CXCL8/Mir-17 clusters [36]. Loss of CD4 + T cells promotes progression to HCC in nonalcoholic fatty liver disease [37] and elimination of neutrophil extracellular traps (NETs) may reduce progression to hepatocellular carcinoma in nonalcoholic steatohepatitis [38]. In our study, we evaluated the connection between ZNF765 and immune cell infiltration and found that there was a positive correlation between the two, especially between ZNF765 and macrophages, CD4+ T cells, and neutrophils mentioned above. Chemokines are small proteins involved in immune cell migration, tumor growth, and immune regulatory dynamics [39, 40], which is why we suspect that they are the key factors mediating ZNF765’s effect on immune cell infiltration. TISIDB and experimental verification revealed that ZNF765 was negatively correlated with CCL28 and that CCL28 expression in shZNF765 cells was significantly lower than in shNC cells, whereas CCL2 and CCL15 were on the contrary. It has been suggested that CCL28 is up-regulated in HCC and can promote the recruitment of regulatory T cells and the invasion and migration of hepatocellular carcinoma [41, 42]. Based on the above results, it is rational to conclude that ZNF765 may influence immune cell infiltration through immune chemokines.

In the TME, T cells can recognize tumor antigens and, at the same time, have a group of cell-surface molecules called immune checkpoints that fine-tune their response [43]. Immune checkpoint inhibitors, including those that target the programmed cell death 1 and programmed cell death ligand 1 (PD-1 and PD-L1) and cytotoxic T lymphocyte antigen 4 (CTLA-4) pathways, are revolutionizing cancer therapy [44, 45]. The association of ZNF765 with T cell checkpoints was evaluated to facilitate the guidance of immunotherapy in patients, as immune checkpoint therapy has been reported to bring benefits to patients with hepatocellular carcinoma [46]. We found that T cell checkpoints CTLA-4, PD-1, and PD-L1 were closely related to ZNF765, and the number of multiple subtypes of T cells may have an effect on the prognosis of patients with hepatocellular carcinoma. To sum up, we believe that ZNF765 may participate in immune infiltration and immune escape in hepatocellular carcinoma patients.

M6-methyladenosine (m^6^A) is an indispensable RNA modification mode [47]. The regulatory factor of m6A modification makes a difference in cancer development and has been shown to participate in the tumorigenesis of HCC [48, 49]. For example, METTL3 can play a carcinogenic role in HCC through YTHDF2-dependent SOCS2 posttranscriptional silencing [50], and stabilization of BLACAT1 expression through RBMX promotes HCC development and drug resistance [51]. Our study focused on the connection between ZNF765 and m^6^A-related genes. The expression of ZNF765 showed the same trend as that of several m^6^A-related genes. Among them, RBMX, METTL3, RBM15B, HNRNPA2B1, and LRPPRC were highly associated with ZNF765, and we also confirmed the regulation of ZNF765 on HNRNPA2B1 and RBMX by western blotting. Additionally, results revealed that up-regulation of these five genes resulted in poor survival in HCC patients and is a risk factor for HCC [52–55]. Consequently, we hypothesized that ZNF765 may promote hepatocellular carcinoma through the m^6^A regulatory factor.

Drug resistance is a major challenge in cancer treatment and can hinder long-term survival [55]. The multi-target tyrosine kinase inhibitor sorafenib, a first-line agent for patients with HCC, has shown resistance in most patients [56]. Accordingly, it makes sense to explore the sensitivity of HCC patients to different drugs. We used ZNF765 and the 4 genes most similar to its expression to explore the drug sensitivity of patients with HCC. The results show that patients with up-regulated ZNF765 expression were sensitive to 20 drugs, especially i-BET-762 and NPK76-ii-72-1, while resistant to docetaxel, 17-AAG, and bleomycin. Further, half maximal inhibitory concentration confirmed the effect of ZNF765 expression on the sensitivity of hepatocellular carcinoma cells to effective docetaxel and bleomycin used in the treatment of patients with hepatocellular carcinoma.

In a nutshell, our study illustrates the fact that ZNF765 may be a potential biomarker that promotes the progression of hepatocellular carcinoma and is associated with a poor prognosis. In our research, ZNF765 may not only affect the cell cycle but also act on the microenvironment of hepatocellular carcinoma to regulate tumor-infiltrating immunity and m^6^A modification and influence patient sensitivity to drugs. At the same time, this study still has some limitations. We started to explore ZNF765 using only the TCGA, ICGC, and HCCDB databases without actual clinical data. Besides, high-quality validation of the above biological functions should be further performed by cell or animal experiments.

## MATERIALS AND METHODS

### Data collection and processing

LIHC gene expression patterns and clinical information are from the TCGA database (https://portal.gdc.cancer.gov) [57] and the ICGC database (https://dcc.icgc.org) [58]. In TCGA, we used 374 cancer samples and 50 normal samples, file type HTSEQ-FPKM, including those for gene expression studies, and clinical information for 377 samples. Meanwhile, 202 normal samples and 243 tumor samples were obtained in the ICGC from the (LINC-JP) Liver Cancer - NCC and JP datasets.

### Patients and tumor specimens

Human HCC samples and matched adjacent samples were obtained from 40 patients undergoing liver resection in the Second Affiliated Hospital of Nanchang University from January 2018 to January 2021. The patient’s informed consent was obtained. At the same time, the research ethics committee of the hospital mentioned above agreed to the experiment.

### Cell culture

Transfection human HCC cell lines LM3 were obtained from the Chinese Academy of Sciences Cell Bank of Type Culture Collection and the Shanghai Institute of Cell Biology in China. The cells were maintained with 5% CO2 at 37°C in DMEM (HyClone, Germany) with 10% fetal bovine serum (GIBCO, USA).

### TIMER database analysis

TIMER (https://cistrome.shinyapps.io/timer), a functional website, could dissect the levels of immune invasion in different kinds of cancer [59]. In our study, the “Diff Exp module” was chosen to recognize ZNF765 expressions in specific types of cancers. Then, the “gene module” was adopted to explore the connection between ZNF765 and immune infiltration in cancer. What’s more, immune cell infiltration levels in various SCNA changes in ZNF765 were compared via the “SCNA module.” The difference between the infiltration level for each SCNA category and the normal was assessed through a two-sided Wilcoxon rank-sum test. Finally, with the help of the “correlation module,” considering Spearman’s rho value (*p* value < 0.05) and predicted statistical implications, the relationship of ZNF765 with the gene markers of immune cells in HCC was proved.

### HCCDB database

HCCDB (http://lifeome.net/database/hccdb), a database designed for exploring HCC, contains 15 public HCC gene expression datasets from 3917 samples [60]. Its data is collected from Gene Expression Omnibus (GEO), the Liver Hepatocellular Carcinoma Project of the Cancer Genome Atlas (TCGA-LIHC), Liver Cancer-RIKEN, and the JP Project from the International Cancer Genome Consortium (ICGC LIRI-JP). We used this website to confirm the high ZNF765 expression in HCC in tumor tissues, and high ZNF765 expression leads to a poor prognosis. A *p* value of 0.05 was considered meaningful.

### UALCAN database analysis

The UALCAN database (http://ualcan.path.uab.edu/) [61] is an oncology data analysis site that provides comprehensive cancer transcriptome and clinical patient data (extracted from TCGA). In our study, the “expression” and “survival” modules from the “Liver Hepatocellular Carcinoma” database were applied to assess the expression and survival of ZNF765, respectively. We also analyzed ZNF765 expression in normal and LIHC samples based on clinicopathological characteristics. We further studied the clinicopathological features of ZNF765 promoter methylation, which include the tumor grade, patient’s gender, age, and others.

### Kaplan-Meier plotter database analysis

We performed a data analysis of ZNF765 expression and survival in 364 HCC patients using Kaplan-Meier Plotter (http://kmplot.com) [62]. The differences in overall survival (OS), progression-free survival (PFS), disease-specific survival (DSS), and relapse-free survival (RFS) were found in patients with HCC receiving different expressions of ZNF765. We also explored the difference in LIHC patients’ survival under different immune cell numbers. Hazard ratios (HRs), 95% confidence intervals (95% CI), and Logrank *p* values were calculated.

### LinkedOmics analysis

LinkedOmics (http://www.linkedomics.org/login.php), a comprehensive online site, is usually chosen to analyze multidimensional data within and across 32 kinds of cancer [63]. Using it, we succeeded in mining the co-expressed genes linked to ZNF765 in the TCGA LIHC database through the results of the analysis. Volcano plots and heat maps provided strong evidence for this. We did a correlation analysis as a concrete measurement of our study. The website was also selected to complete the GO term and KEGG pathway enrichment analysis. It was aimed at identifying the Gene Ontology (GO) annotations and pathways. Pathways with *p* value < 0.05 were considered as the standard.

### Gene set enrichment analysis (GSEA)

Usually, Gene Set Enrichment Analysis (GSEA) is adopted for genome-wide expression profile analysis and interpretation built on biological knowledge [64]. The RNA-seq data of 374 HCC cases were downloaded from Genomic Data Commons (https://portal.gdc.cancer.gov/). We set each HCC patient’s ZNF765 expression into two expression groups. The parameters were established, such as gene set database: h. All. V7.4 Symbols. gmt (Hallmarks); number of permutations: 1,000. *p* value < 0.05 and a false discovery rate (FDR) < 0.25 were considered as meaningful.

### PPI network construction

We applied the PPI network of the STRING website (https://string-db.org/) [65] to do research about the connection between the 500 most related genes. The parameter of medium confidence was set at 0.4. The top 500 genes were evaluated by Cytoscape 3.8.2 and its plug-in MCODE (Molecular Complex Detection). The selection criteria are as follows: Max depth = 100, node score cutoff = 0.2, K-core = 2.

### GeneMANIA analysis

GeneMANIA (http://www.genemania.org) is a powerful website for gene analysis with its large and interwoven data network [66]. We chose it to build an interactive functional network of ZNF765 in HCC. We can get a lot from its functional network, for example, physical interaction, gene co-expression, gene co-localization, gene enrichment analysis, and website prediction. In the network, we used different kinds of circles of different sizes to represent the protein, and the number and thickness of the connection indicated the strength of the connection between the two sides of the connection.

### GEPIA analysis

GEPIA (http://gepia.cancer-pku.cn/) [67] is an analysis site using the TCGA and GTEx databases. In our study, the TCGA LIHC database was applied to evaluate the expression of ZNF765. In GEPIA’s “Expression DIY” module, log2 (TPM+1) was used for logarithmic scale matching to assess the expression difference of ZNF765 between LIHC and normal adjacent LIHC tissue samples. In addition, the Spearman coefficient was used to analyze the correlation between ZNF765 and PD-1, PD-L1, and CTLA-4 in the “correlation analysis” module. In the “Survival” module, we plotted the overall survival situation of m^6^A-related genes, HNRNPA2B1, METTL3, RBMX, RBM15B, and LRPPRC, respectively.

### TISIDB analysis

TISIDB (http://cis.hku.hk/TISIDB) integrates multiple types of data resources from tumor immune technology to study tumor immune interactions [68]. The “Chemokine” module was used to analyze Spearman correlations between ZNF765 and chemokines, CCL2, CCL15, and CCL28, across liver hepatocellular carcinoma (LIHC).

### Cancer pathway activity and drug sensitivity

GSCALite (http://bioinfo.life.hust.edu.cn/web/GSCALite/) integrates large amounts of multi-omics and drug data to assess a series of genes in cancer [69]. The genes with the highest correlation with ZNF765 expression, ZNF587, ZNF611, ZNF761, and ZNF845, were used for the “Pathway Activity” and “Drug Sensitivity” module analyses in the TCGA-LIHC dataset of the GSCALite website.

### Correlation analysis

We performed correlation analysis of gene expression using R software (limma, ggplot2, and pheatmap packages). We plotted heat maps to illustrate the correlation between ZNF765 and m^6^A-related genes. The relationships among ZNF765 and the most relevant five genes were visualized in scatter diagrams.

### Immunohistochemistry

Initially, the LIHC tissue and adjacent tissues were fixed in formalin and embedded in paraffin, and cut into 4-um-thick sections [70]. After the antigen was repaired by deparaffinization, rehydration, and microwave heating in a microwave-heated antigenic unsealing solution (EDTA, pH 8.0), the slices were sealed with goat serum for 30 minutes. The sections are then incubated at 4°C overnight with anti-ZNF765 monoclonal antibody (ZNF765-Biorbyt-Catalog number: orb472218). Afterward, the HRP-conjugated secondary antibody (Boster) was placed at room temperature for 2 h. Subsequently, the two-step approach (catalog no: PV-9000; ZSGB-BIO Co., Ltd., Beijing, China) was used for immunostaining. Ultimately, three pathologists who are unfamiliar with the clinical parameters appraised the staining intensity and the proportion of positive cells semi-quantitatively.

### Colony formation assay

The transfected cells were cultured for 48 h, and then a total of 3 × 10^3^ cells were cultured in 6-well plates. Ten days later, 4% paraformaldehyde was used to fix cells for 35 minutes. Then, they were stained with 1.0% crystal violet for 30 min, until visible clones form. The number of colonies was counted in 10 different fields.

### Transwell assay

Human HCCLM3 cells were inoculated into the upper chamber with a serum-free medium. Each well was a density of 2 × 10^6^ cells. The bottom chamber is filled with 500 μl of 20% fetal bovine serum (FBS) culture medium. After incubating in a 5% (v/v) CO2 incubator at RT for 2 d, and removing the non-invasive cells and matrigel in the upper chamber, the cells were fixed on the lower surface with 10% neutral buffered formalin solution and 0.1% crystal violet staining. The invading cells in five randomly selected microscope fields were counted.

### Real-time RT-PCR analyses

Total mRNA was extracted with the standard Trizol-based protocol (Invitrogen, USA), and PrimeScript RT Reagent Kit (Invitrogen, USA) performed a reverse transcription reaction. The qPCR was conducted by SYBR Premix Ex Taq (TaKaRa, China). Finally, a semi-quantitative analysis was performed. This technique was adopted in our study to examine the relative mRNA expression of CCL2, CCL15, and CCL28.

### Western blot

Total protein was obtained from LIHC cells using the RIPA protein assay (Beyotime, Shanghai, China). After centrifugation, the concentration of protein was measured by the BCA protein assay kit (Thermo Scientific, Waltham, MA, USA). Then, the quantities of protein (40 ug) were loaded onto SDS-PAGE electrophoreses and transferred to the PVDF membrane. Having incubated with the indicated antibodies overnight at 4°C, the membrane was washed three times with TBST. Then, the membranes were incubated with ZNF765-, HNRNPA2B1-, or RBMX-conjugated secondary antibodies for 1 h at room temperature. Finally, the membranes were observed by using an enhanced chemiluminescence (ECL) kit.

### Half-maximal inhibitory concentration (IC50)

The HCCLM3 cells were treated with serially diluted docetaxel and bleomycin via a Janus automated workstation (PerkinElmer). Analysis of cell viability was done using a firefly-luciferase based ATP monitoring system (ATPLite™ 1step; PerkinElmer). Half maximal inhibitory concentration values were determined by performing logarithm-normalized sigmoidal dose curve fitting using the GraphPad Prism 6 software.

### Statistical analysis

All statistical analysis in this work was done by R software (version 4.1.2). The detection of different ZNF765 expression levels between LIHC samples and normal samples was realized by using the “limma” and “beeswarm” packages of “R” and the rank sum test method. The connection between ZNF765 and clinical characteristics was evaluated by the Wilcoxon signed-rank test or Kruskal-Wallis test and logistic regression. The Kaplan-Meier curve was then drawn using the log-rank test to investigate prognosis distribution among patients with different expressions to determine whether the differential expression of genes played an important role in patient survival (*p* < 0.05). Univariate and multivariate Cox regression analysis identified factors significantly associated with prognosis (*p* < 0.05) (the Cox model uses the “survival” and “survminer” packages of “R”). Lastly, the ROC curve drawn by the “survival ROC” was applied to analyze the predictive capacity of the ZNF765 expression level over one, three, or five years.

## Supplementary Materials

Supplementary Figures

Supplementary Tables
