# Supplementary material for: ZNF765 is a prognostic biomarker of hepatocellular carcinoma associated with cell cycle, immune infiltration, m6A modification, and drug susceptibility
Source: Aging (Albany NY). 2023 Jul 4;15(13):6179–211. doi: 10.18632/aging.204827 (PMC10373972; doi:10.18632/aging.204827)
Supplement: Supplementary Figures [file aging-15-204827-s001.pdf]

## SUPPLEMENTARY FIGURES

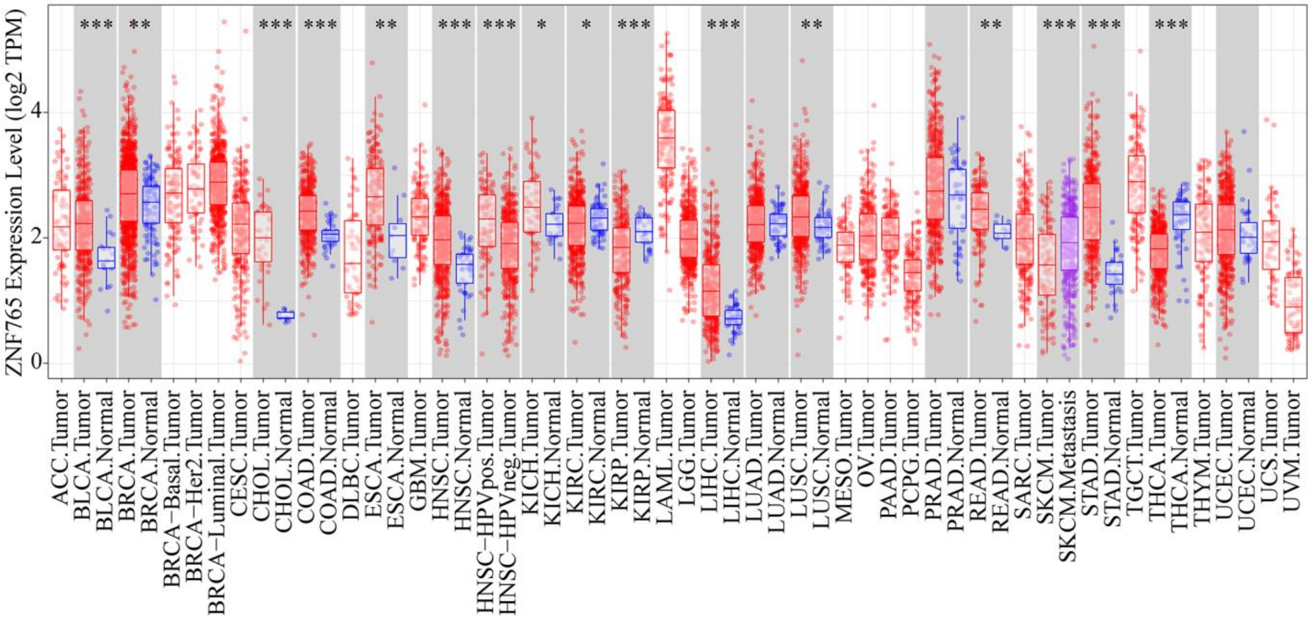


**Supplementary Figure 1. The expression of ZNF765 in pan-cancers.** Level of ZNF765 expression in a variety of cancer tissues (data from the TIMER database) (***$p < 0.001$, **$p < 0.01$, *$p < 0.05$).

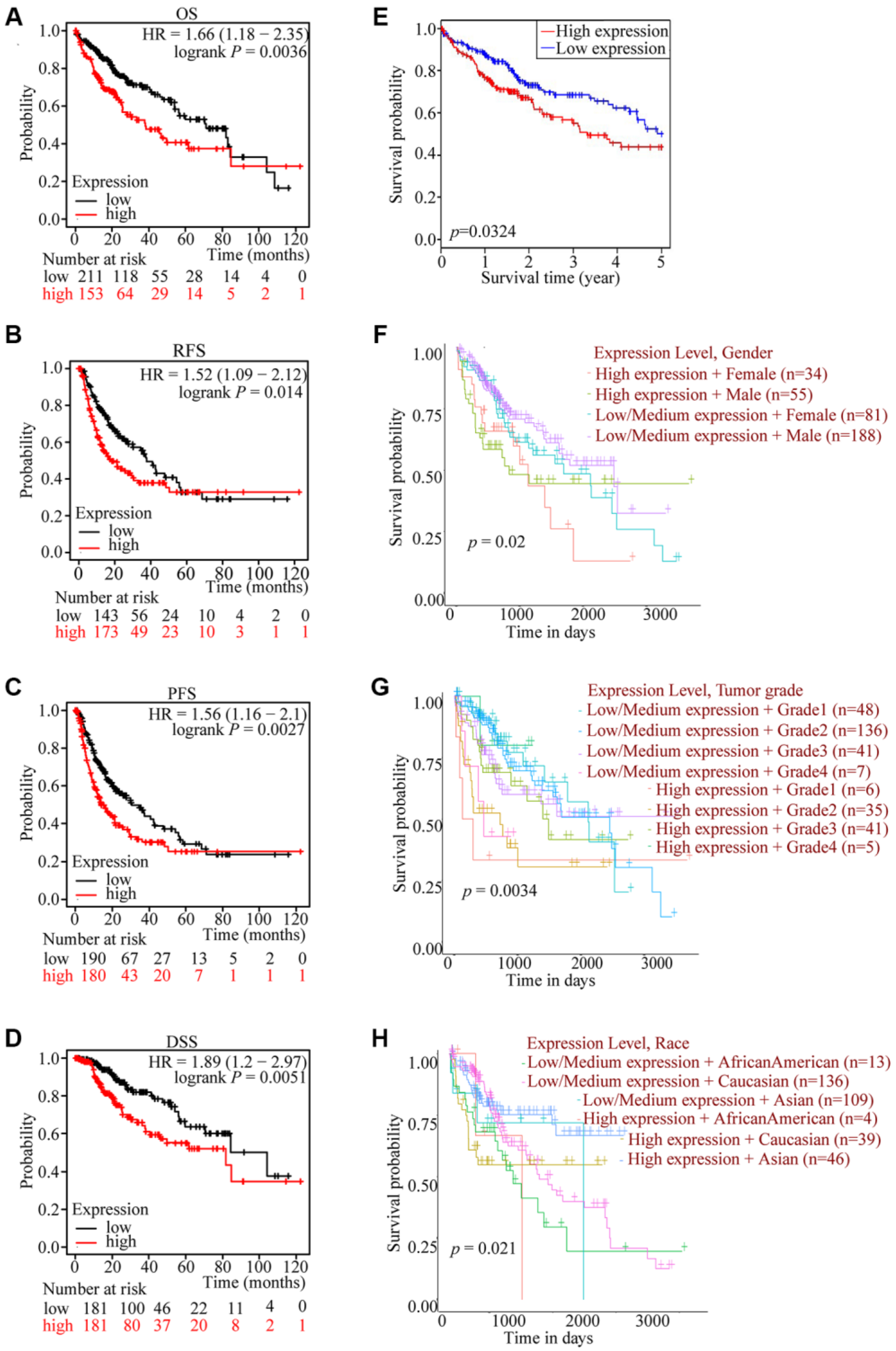


**Supplementary Figure 2. The function of high ZNF765 expression in prognosis.** (**A**–**D**) Kaplan-Meier analysis of OS (overall survival), RFS (relapse-free survival), PFS (progression-free survival), and DSS (disease-specific survival) in the HCC patients. (**E**) Effect of ZNF765 mRNA expression level on HCC patient survival by HCCDB ($p$ = 0.0324). (**F**) Survival probability of HCC patients with different genders and ZNF765 expression. (**G**) Survival probability of HCC patients with different tumor grades and ZNF765 expression. (**H**) Survival probability of HCC patients between different races and ZNF765 expression.

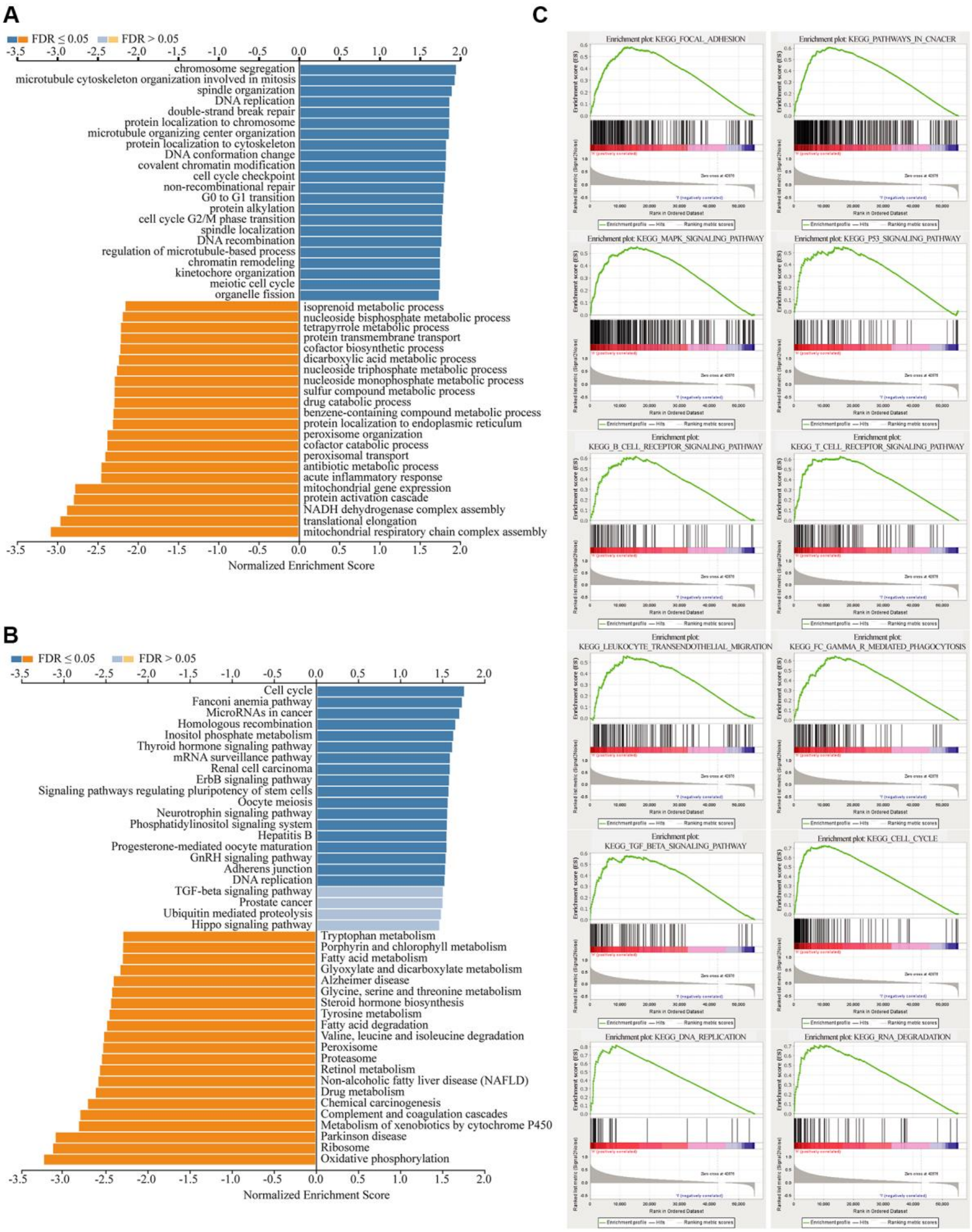


**Supplementary Figure 3. Enrichment analysis of ZNF765 functional networks in HCC.** (**A**) GO pathway analysis. Dark blue and orange indicate an FDR ≤ 0.05, whereas light blue and orange indicate an FDR greater than 0.05. FDR, false discovery rate. (**B**) KEGG pathway analysis. FDR ≤ 0.05 is indicated by dark blue and orange; FDR > 0.05 is indicated by light blue and orange. (**C**) GSEA analysis of ZNF765 based on expression in the TCGA-LIHC dataset. Abbreviation: NES: normalized enrichment score. NOM *p*-value: nominal *p* value; FDR *q*-val: false discovery rate.

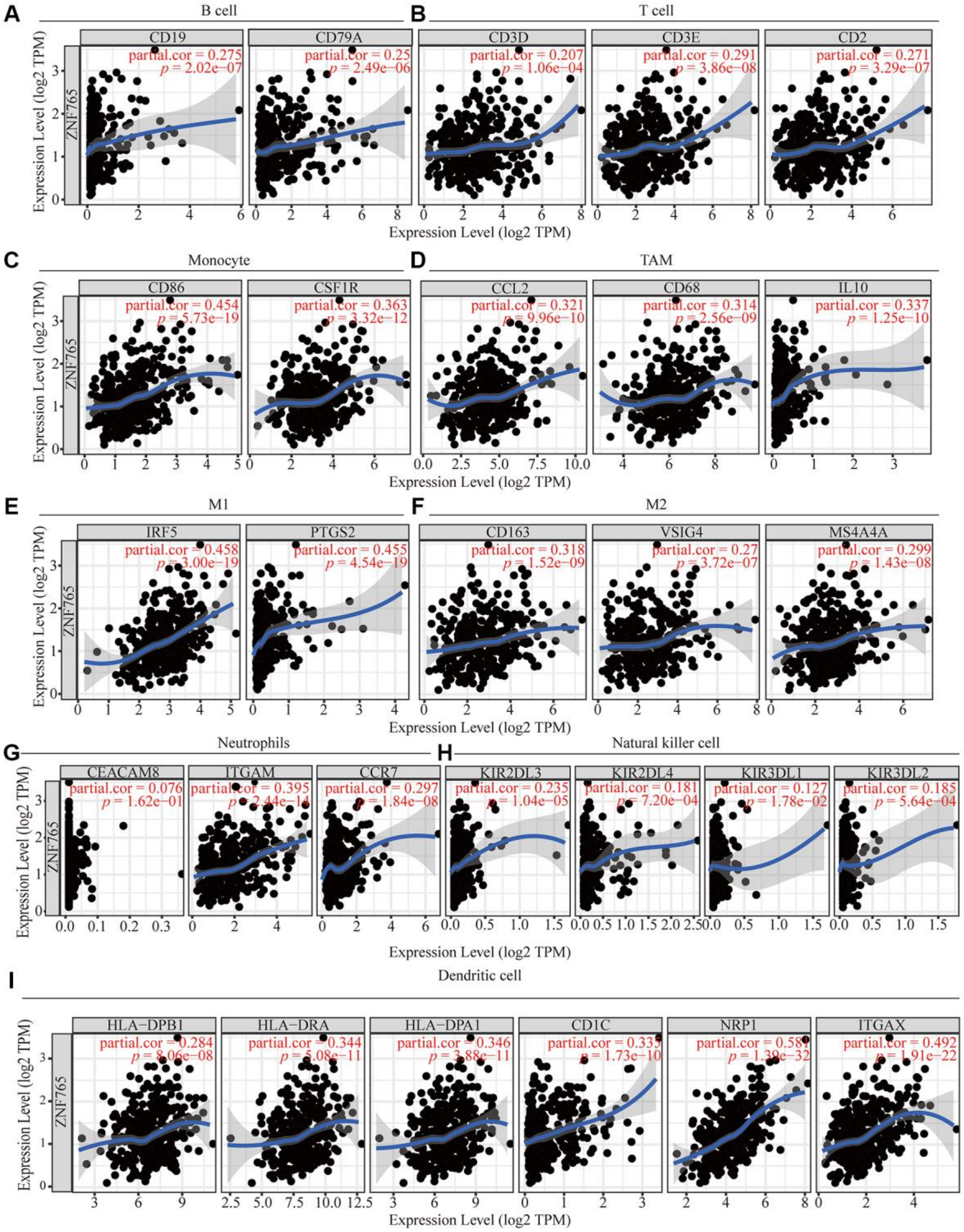


**Supplementary Figure 4. Correlation of ZNF765 expression and the marker genes of infiltrating immune cells in HCC (TIMER).** The scatter plots showed correlation between ZNF765 expression and the gene markers of (**A**) B cell (CD79A, CD19); (**B**) T cell (CD3D, CD3E, CD2); (**C**) Monocyte (CD86, CSF1R); (**D**) TAM cell (CCL2, CD68, IL10); (**E**) M1 cell (IRF5, PTGS2); (**F**) M2 cell (CD163, VSIG4, MS4A4A); (**G**) Neutrophils (ITGAM, CCR7, CEACAM8); (**H**) Natural killer cell (KIR2DL3, KIR2DL4, KIR3DL1, KIR3DL2); (**I**) Dendritic cell (HLA-DPB1, HLA-DRA, HLA-DPA1, CD1C, NRP1, ITGAX).

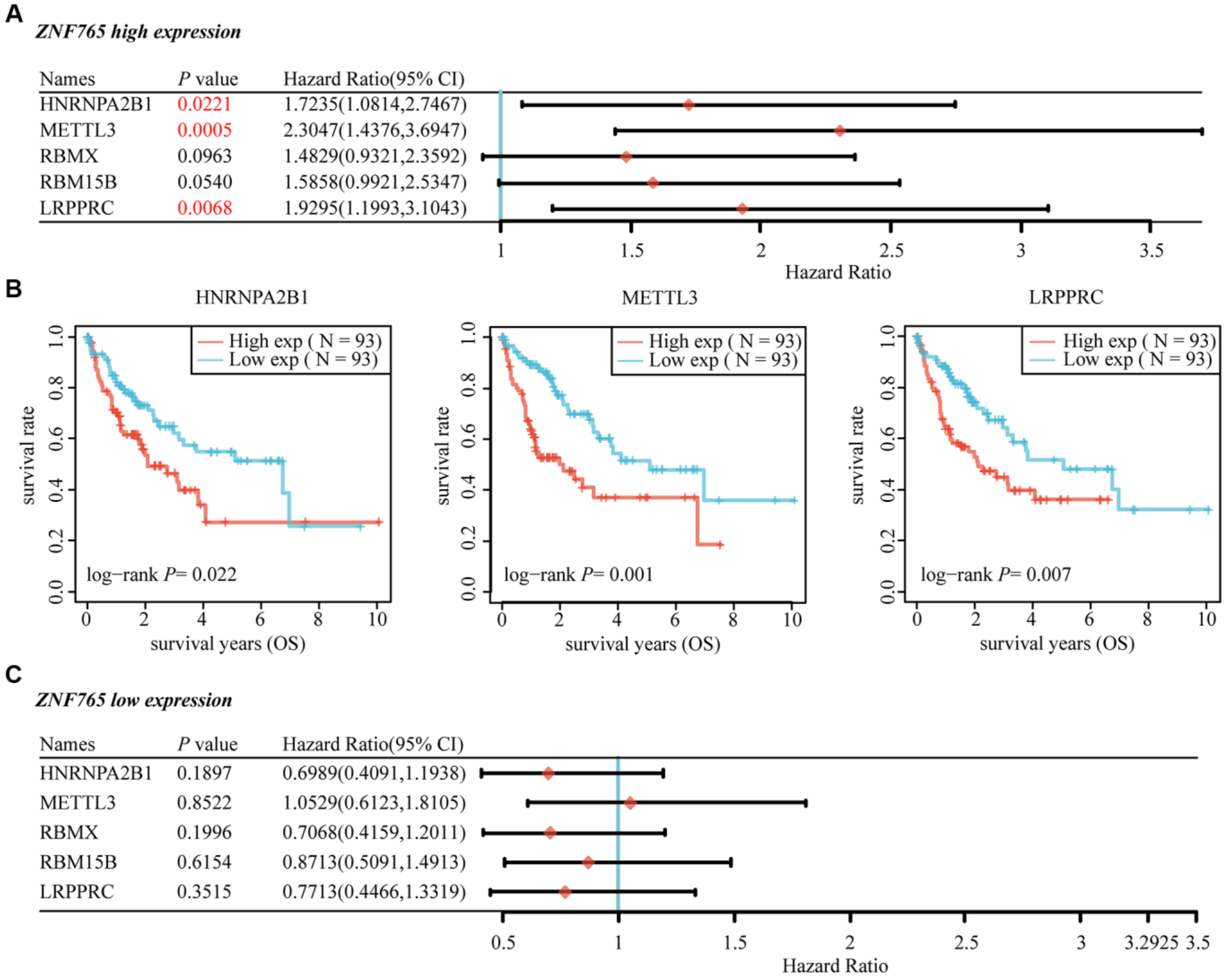


**Supplementary Figure 5. ZNF765 affects the prognosis of HCC patients by affecting some m6A regulators.** (**A**) HNRNPA2B1, METTL3, and LRPPRC were risk factors for HCC when ZNF765 was highly expressed. (**B**) When ZNF765 was highly expressed, high expression of HNRNPA2B1, METTL3 or LRPPRC led to poor survival in HCC patients. (**C**) HNRNPA2B1, METTL3, and LRPPRC had no effect on the prognosis of HCC patients when ZNF765 was lowly expressed.
