# Supplementary material for: ZNF765 is a prognostic biomarker of hepatocellular carcinoma associated with cell cycle, immune infiltration, m6A modification, and drug susceptibility
Source: Aging (Albany NY). 2023 Jul 4;15(13):6179–211. doi: 10.18632/aging.204827 (PMC10373972; doi:10.18632/aging.204827)
Supplement: Supplementary Tables [file aging-15-204827-s002.pdf]

SUPPLEMENTARY TABLES

Supplementary Table 1. Univariate and multivariate COX regression analysis of factors associated with OS in HCC patients.

Variable	Univariate analysis			Multivariate analysis		
	HR	95% CI	*p* value	HR	95% CI	*p* value
Age	1.007	0.990–1.024	0.441	1.018	0.999–1.038	0.069
Gender	0.839	0.536–1.314	0.443	1.198	0.735–1.953	0.469
Grade	1.073	0.795–1.449	0.645	1.098	0.795–1.518	0.57
Stage	1.809	1.426–2.294	**<0.001**	0.928	0.396–2.172	0.863
T	1.767	1.415–2.207	**<0.001**	1.805	0.828–3.936	0.137
M	3.892	1.223–12.386	**0.021**	2.079	0.556–7.777	0.277
ZNF765	3.728	1.775–7.830	**0.001**	4.314	1.815–10.253	**0.001**

Abbreviations: OS: overall survival; HR: hazard ratio; CI: confidence interval; T: tumor; N: node; M: metastasis; Bold values indicate *p* values <0.05.

Supplementary Table 2. Relationship between ZNF765 and gene markers of different immune cells using the TIMER database.

Description	Gene markers	LIHC			
		None		Purity	
		Cor	*p*	Cor	*p*
**B cell**	CD19	0.218	**2.23E-05**	0.275	**2.02E-07**
	CD79A	0.154	**3.01E-03**	0.25	**2.49E-06**
**T cell (general)**	CD3D	0.109	**3.55E-02**	0.207	**1.06E-04**
	CD3E	0.155	**2.75E-03**	0.291	**3.86E-08**
	CD2	0.146	**4.74E-03**	0.271	**3.29E-07**
**CD8+ T cell**	CD8A	0.171	**9.53E-04**	0.272	**3.02E-07**
	CD8B	0.077	1.39E-01	0.16	**2.83E-03**
**Monocyte**	CD86	0.306	**2.12E-09**	0.454	**5.73E-19**
	CSF1R	0.222	**1.67E-05**	0.363	**3.32E-12**
**TAM**	CCL2	0.196	**1.54E-04**	0.321	**9.96E-10**
	CD68	0.221	**1.90E-05**	0.314	**2.56E-09**
	IL10	0.24	**2.99E-06**	0.337	**1.25E-10**
**M1**	IRF5	0.464	**3.53E-21**	0.458	**3.00E-19**
	PTGS2	0.307	**1.47E-09**	0.455	**4.54E-19**
**M2**	CD163	0.204	**7.29E-05**	0.318	**1.52E-09**
	VSIG4	0.161	**1.94E-03**	0.27	**3.72E-07**
	MS4A4A	0.169	**1.07E-03**	0.299	**1.43E-08**
**Neutrophils**	CEACAM8	0.051	3.24E-01	0.076	**1.62E-01**
	ITGAM	0.301	**4.08E-09**	0.395	**2.44E-14**
	CCR7	0.17	**1.04E-03**	0.297	**1.84E-08**

**Natural killer cell**	KIR2DL1	0.06	2.48E-01	0.048	3.75E-01
	KIR2DL3	0.194	**1.73E-04**	0.235	**1.04E-05**
	KIR2DL4	0.158	**2.27E-03**	0.181	**7.20E-04**
	KIR3DL1	0.113	**2.91E-02**	0.127	**1.78E-02**
	KIR3DL2	0.134	**9.70E-03**	0.185	**5.64E-04**
	KIR3DL3	0.031	5.53E-01	0.018	7.43E-01
**Dendritic cell**	HLA-DPB1	0.177	**6.19E-04**	0.284	**8.06E-08**
	HLA-DQB1	0.101	5.27E-02	0.192	**3.45E-04**
	HLA-DRA	0.229	**8.92E-06**	0.344	**5.08E-11**
	HLA-DPA1	0.222	**1.62E-05**	0.346	**3.88E-11**
	CD1C	0.242	**2.41E-06**	0.335	**1.73E-10**
	NRP1	0.529	**0.00E+00**	0.581	**1.39E-32**
	ITGAX	0.368	**3.50E-13**	0.492	**1.91E-22**

Supplementary Table 3. Correlation analysis between ZNF765 and gene markers of different types of T cells in TIMER.

**Description**	**Gene markers**	**LIHC**			
		**None**		**Purity**	
		**Cor**	***p***	**Cor**	***p***
**Th1**	TBX21	0.143	**5.87E-03**	0.239	**6.93E-06**
	STAT4	0.221	**1.90E-05**	0.284	**7.69E-08**
	STAT1	0.484	**0.00E+00**	0.531	**1.69E-26**
	TNF	0.295	**7.21E-09**	0.406	**3.93E-15**
	IFNG	0.171	**9.32E-04**	0.243	**5.20E-06**
**Th1-like**	HAVCR2	0.302	**3.50E-09**	0.454	**6.21E-19**
	IFNG	0.171	**9.32E-04**	0.243	**5.20E-06**
	CXCR3	0.187	**2.95E-04**	0.282	**1.01E-07**
	BHLHE40	0.404	**0.00E+00**	0.427	**1.08E-16**
	CD4	0.269	**1.62E-07**	0.35	**2.17E-11**
**Th2**	STAT6	0.39	**6.88E-15**	0.38	**2.88E-13**
	STAT5A	0.35	**3.96E-12**	0.423	**2.02E-16**
**Treg**	FOXP3	0.247	**1.46E-06**	0.298	**1.60E-08**
	CCR8	0.472	**6.08E-22**	0.571	**3.18E-31**
	TGFB1	0.341	**1.99E-11**	0.448	**1.84E-18**
**Resting Treg**	FOXP3	0.247	**1.46E-06**	0.298	**1.60E-08**
	IL2RA	0.281	**3.57E-08**	0.408	**2.99E-15**
**Effector Treg T-cell**	FOXP3	0.247	**1.46E-06**	0.298	**1.60E-08**
	CCR8	0.472	**6.08E-22**	0.571	**3.18E-31**
	TNFRSF9	0.4	**1.05E-15**	0.501	**2.56E-23**
**Effector T-cell**	CX3CR1	0.475	**0.00E+00**	0.529	**2.91E-26**
	FGFBP2	0.008	8.72E-01	0.039	4.66E-01
	FCGR3A	0.295	**8.36E-09**	0.389	**7.09E-14**

**Naïve T-cell**	CCR7	0.17	**1.04E-03**	0.297	**1.84E-08**
	SELL	0.296	**6.89E-09**	0.421	**3.05E-16**
**Effector memory T-cell**	DUSP4	0.341	**2.06E-11**	0.445	**3.64E-18**
	GZMK	0.084	1.07E-01	0.187	**4.93E-04**
	GZMA	0.065	2.11E-01	0.153	**4.41E-03**
**Resident memory T-cell**	CD69	0.259	**4.21E-07**	0.396	**2.11E-14**
	CXCR6	0.164	**1.57E-03**	0.29	**4.21E-08**
	MYADM	0.604	**0.00E+00**	0.647	**2.96E-42**
**General**	CCR7	0.17	**1.04E-03**	0.297	**1.84E-08**
**memory T-cell**	SELL	0.296	**6.89E-09**	0.421	**3.05E-16**
	IL7R	0.33	**6.88E-11**	0.469	**3.17E-20**
**Exhausted T-cell**	HAVCR2	0.302	**3.50E-09**	0.454	**6.21E-19**
	LAG3	0.154	**3.03E-03**	0.181	**7.33E-04**
	CXCL13	0.164	**1.52E-03**	0.222	**3.04E-05**
	LAYN	0.348	**6.73E-12**	0.442	**6.46E-18**
